# Biological and clinical characteristics of gene carriers far from predicted onset in the Huntington's disease Young Adult Study (HD-YAS): a cross-sectional analysis

**DOI:** 10.1016/S1474-4422(20)30143-5

**Published:** 2020-06

**Authors:** Rachael I Scahill, Paul Zeun, Katherine Osborne-Crowley, Eileanoir B Johnson, Sarah Gregory, Christopher Parker, Jessica Lowe, Akshay Nair, Claire O'Callaghan, Christelle Langley, Marina Papoutsi, Peter McColgan, Carlos Estevez-Fraga, Kate Fayer, Henny Wellington, Filipe B Rodrigues, Lauren M Byrne, Amanda Heselgrave, Harpreet Hyare, Cristina Sampaio, Henrik Zetterberg, Hui Zhang, Edward J Wild, Geraint Rees, Trevor W Robbins, Barbara J Sahakian, Douglas Langbehn, Sarah J Tabrizi

**Affiliations:** a Huntington's Disease Centre, Department of Neurodegenerative disease, UCL Queen Square Institute of Neurology, University College London, London, UK; b Division of Equity, Diversity and Inclusion, University of New South Wales, Sydney, NSW, Australia; c Department of Computer Science and Centre for Medical Image Computing, University College London, London, UK; d Max Planck University College London Centre for Computational Psychiatry and Ageing Research, UCL Queen Square Institute of Neurology, London, UK; e Department of Psychiatry and Behavioural and Clinical Neuroscience Institute, University of Cambridge, Cambridge, UK; f Brain and Mind Centre, University of Sydney, Sydney, NSW, Australia; g Department of Neurodegenerative Disease, UCL Queen Square Institute of Neurology, University College London, London, UK; h Dementia Research Institute at University College London, London, UK; i Department of Brain Repair and Rehabilitation, University College London Institute of Neurology, London, UK; j CHDI Foundation, Princeton, NJ, USA; k Instituto de Medicina Molecular, Faculdade de Medicina de Lisboa, Lisbon, Portugal; l Clinical Neurochemistry Laboratory, Sahlgrenska University Hospital, Mölndal, Sweden; m Department of Psychiatry and Neurochemistry, Institute of Neuroscience and Physiology, Sahlgrenska Academy at University of Gothenburg, Mölndal, Sweden; n University College London Institute of Cognitive Neuroscience, University College London, London, UK; o Department of Psychology and Behavioural and Clinical Neuroscience Institute, University of Cambridge, Cambridge, UK; p Department of Psychiatry, University of Iowa, Iowa City, IA, USA

## Abstract

**Background:**

Disease-modifying treatments are in development for Huntington's disease; crucial to their success is to identify a timepoint in a patient's life when there is a measurable biomarker of early neurodegeneration while clinical function is still intact. We aimed to identify this timepoint in a novel cohort of young adult premanifest Huntington's disease gene carriers (preHD) far from predicted clinical symptom onset.

**Methods:**

We did the Huntington's disease Young Adult Study (HD-YAS) in the UK. We recruited young adults with preHD and controls matched for age, education, and sex to ensure each group had at least 60 participants with imaging data, accounting for scan fails. Controls either had a family history of Huntington's disease but a negative genetic test, or no known family history of Huntington's disease. All participants underwent detailed neuropsychiatric and cognitive assessments, including tests from the Cambridge Neuropsychological Test Automated Battery and a battery assessing emotion, motivation, impulsivity and social cognition (EMOTICOM). Imaging (done for all participants without contraindications) included volumetric MRI, diffusion imaging, and multiparametric mapping. Biofluid markers of neuronal health were examined using blood and CSF collection. We did a cross-sectional analysis using general least-squares linear models to assess group differences and associations with age and CAG length, relating to predicted years to clinical onset. Results were corrected for multiple comparisons using the false discovery rate (FDR), with FDR <0·05 deemed a significant result.

**Findings:**

Data were obtained between Aug 2, 2017, and April 25, 2019. We recruited 64 young adults with preHD and 67 controls. Mean ages of participants were 29·0 years (SD 5·6) and 29·1 years (5·7) in the preHD and control groups, respectively. We noted no significant evidence of cognitive or psychiatric impairment in preHD participants 23·6 years (SD 5·8) from predicted onset (FDR 0·22–0·87 for cognitive measures, 0·31–0·91 for neuropsychiatric measures). The preHD cohort had slightly smaller putamen volumes (FDR=0·03), but this did not appear to be closely related to predicted years to onset (FDR=0·54). There were no group differences in other brain imaging measures (FDR >0·16). CSF neurofilament light protein (NfL), plasma NfL, and CSF YKL-40 were elevated in this far-from-onset preHD cohort compared with controls (FDR<0·0001, =0·01, and =0·03, respectively). CSF NfL elevations were more likely in individuals closer to expected clinical onset (FDR <0·0001).

**Interpretation:**

We report normal brain function yet a rise in sensitive measures of neurodegeneration in a preHD cohort approximately 24 years from predicted clinical onset. CSF NfL appears to be a more sensitive measure than plasma NfL to monitor disease progression. This preHD cohort is one of the earliest yet studied, and our findings could be used to inform decisions about when to initiate a potential future intervention to delay or prevent further neurodegeneration while function is intact.

**Funding:**

Wellcome Trust, CHDI Foundation.

## Introduction

Huntington's disease is an autosomal dominant neurodegenerative condition caused by a CAG expansion in the *HTT* gene, resulting in the expression of mutant huntingtin protein, which is thought to be the predominant toxic agent. Clinically, Huntington's disease is characterised by gradual deterioration of motor and cognitive function and neuropsychiatric disturbance. In an era of new therapies capable of targeting DNA and RNA, the known single genetic cause of Huntington's disease provides an attractive target for such treatments, with several huntingtin-lowering drugs now in human trials.^1^, ^2^ An appropriate time to initiate therapy in a preventive trial would be before clinical functioning has been affected, but when one or more measurable biomarkers of neurodegeneration can be used for enrichment or stratification and to monitor efficacy.

**Research in context**

**Evidence before this study**

We reviewed the scientific literature on the premanifest phase of Huntington's disease (preHD), searching PubMed on Dec 17, 2019, with no restrictions on start date or language, for articles published in the previous 10 years with human adult participants and the following search terms: “Huntington disease” [MeSH] AND (“prodromal” OR “premanifest” OR “presymptomatic” [Title/Abstract]). Our search yielded 931 studies. We found robust evidence of disease-related differences across multiple modalities in preHD up to 15 years before expected clinical disease onset. Such differences include increases in biofluid biomarkers of neuronal damage, brain atrophy focused in the subcortical structures accompanied by involvement of white matter networks, and subtle impairment in cognition, motor function, and neuropsychiatry. We are not aware of studies that include a comprehensive, multimodal assessment of adult premanifest gene carriers more than 20 years from predicted onset.

**Added value of this study**

Our study presents comprehensive phenotyping of a preHD cohort who were on average approximately 24 years from predicted onset, compared with a closely matched control group. Assessments included detailed cognitive, neuropsychiatric, and biofluid biomarker (plasma and CSF) assessments and state of the art volumetric and diffusion imaging. For the first time in studies of Huntington's disease, we also assessed multiparametric imaging, providing brain estimates of myelin and iron, as well as CSF measurements of total huntingtin. We noted no detectable motor, cognitive, or psychiatric differences in preHD at this stage. Brain imaging did not reveal any significant differences in caudate, white matter, or cortical grey matter volumes, and there were also no differences in diffusion and multiparametric mapping measures. There were, however, elevations in CSF mutant huntingtin, neurofilament light protein (NfL), YKL-40, and plasma NfL in individuals with preHD, alongside reduced putamen volumes. CSF NfL showed the strongest effect size of all measures and was the only measure associated with predicted years to onset in this cohort, with higher values in individuals closer to predicted onset. 53% of individuals with preHD had CSF NfL values in the normal range, suggesting that this biomarker first becomes abnormal approximately 24 years from predicted onset. Although CSF mutant huntingtin was detectable at low concentrations in individuals with preHD, most of these individuals had values below the limit of quantification. Total huntingtin concentrations were unchanged in individuals with preHD.

**Implications of all the available evidence**

With several potential disease-modifying treatments in development for Huntington's disease, including mutant and total huntingtin-lowering approaches, identifying the optimum time to treat and suitable biomarkers for trials in early preHD is of timely importance. Our findings suggest that approximately 24 years from predicted onset, when cognitive and neuropsychiatric function appears intact, represents a potentially appropriate time to initiate future disease-modifying therapies. In such a trial, CSF NfL seems as though it would be the most suitable biomarker to monitor progression and, eventually, efficacy, showing superior sensitivity than plasma NfL when measured far from disease onset contrary to closer to disease onset, when sensitivity of CSF and plasma NfL have near equivalence. Although putamen volume is also reduced at this stage, the smaller effect size and absence of a strong association with predicted years to onset could limit its use in clinical trials in early preHD. Suppression of CSF mutant huntingtin to undetectable concentrations could be a viable measure of target engagement for such trials. However, because concentrations of mutant huntingtin are frequently only just above the detection limit, total huntingtin concentrations could be used to provide a measure of percentage huntingtin reduction for total huntingtin-lowering trials. These results are likely to have a major effect on the direction and design of future clinical trials in Huntington's disease.

A detailed characterisation of the premanifest period in Huntington's disease is crucial for disease staging, informing the optimum time to initiate treatments, and identifying biomarkers for future trials in people with premanifest Huntington's disease (preHD). Although the most appropriate markers in the earliest premanifest period are unknown, the genetic basis of Huntington's disease offers the potential to identify this treatment window. First, with complete penetrance, it is possible to identify preHD via genetic testing before clinical onset—ie, the emergence of clear motor manifestations of the disease. Second, there are well-established models to estimate the time to expected clinical onset by using the strong influence of CAG expansion length on age of onset.^3^ These models have shown clear relationships between estimated years to clinical onset and several biomarkers of neurodegeneration.^4^

Multisite observational studies in preHD such as TRACK-HD,^4^ PREDICT-HD,^5^ and ENROLL-HD^6^ have consistently reported subtle motor, cognitive, and neuropsychiatric impairments at least 10–15 years before clinical onset. At this stage, Huntington's disease pathology has already had a widespread effect on brain structure, with extensive evidence of striatal atrophy and white matter degeneration.^4^, ^5^ Elevations in plasma neurofilament light protein (NfL) a marker of axonal damage, were noted in the TRACK-HD cohort^7^ and such biofluid markers might be among the earliest detectable alterations in Huntington's disease.^8^ Because the preHD cohorts studied to date already show disease effects across these many domains, if we are to identify the earliest manifestations of Huntington's disease pathology and establish whether there is a time when they are undetectable, we need to look back to even earlier in the disease process.

Using state-of-the-art methods, we examined potential group differences across multiple domains between healthy controls and a preHD cohort far from predicted onset. We aimed to assess how early disease-related changes can be identified (ie, when there is a measurable biomarker of early neurodegeneration but clinical function is still intact), and which measures are most sensitive in early preHD.

## Methods

### Study design and participants

For the Huntington's disease Young Adult Study (HD-YAS) we recruited preHD gene carriers and controls from across the UK. PreHD participants required a previous positive Huntington's disease genetic test (CAG ≥40) but without showing clinical signs of the disease: all had a Unified Huntington's Disease Rating Scale Total Motor Score (UHDRS TMS)^9^ of 5 or less. Exclusion criteria included contraindication to MRI scanning, significant comorbidity, at-risk genetic status (ie, someone who has not had a genetic test but who has a family history of Huntington's disease), and reduced penetrance CAG repeat length (ie, 36–39). Disease Burden Score,^10^ a product of age and CAG length, was required to be 240 or less, which approximates to more than 18 years from predicted clinical onset. CAG lengths were re-measured at a single lab for statistical analysis. Controls were gene negative (ie, having a family history of Huntington's disease but a negative genetic test), family members with no Huntington's disease risk (eg, partners or spouses of gene carriers), or members of the wider Huntington's disease community (recruited through support groups or as friends of participants). Gene carriers and controls were matched by monitoring group means for sex, age, years of education, and SD for age and education as recruitment progressed, to aid targeted recruitment. The study was approved by the Bloomsbury Research Ethics Committee and all participants gave written informed consent prior to study entry.

### Outcomes and procedures

All outcomes by modality are listed in the panel. All participants underwent extensive assessment of cognitive and neuropsychiatric function, neuroimaging, blood sampling, and optional CSF collection; full details are in the appendix (pp 6–17), and a list is provided in the panel.

Panel **Assessments in the Huntington's disease Young Adult Study**

**Cognition**

*CANTAB*

• Intra-extra dimensional set shift
• Paired associate learning
• Rapid visual processing
• One touch stockings of Cambridge
• Spatial working memory
• Stop signal task

*EMOTICOM*

• Emotion intensity face morphing—increasing and decreasing
• Moral judgement
• Progressive ratio

*Other*

• Stroop colour
• Stroop word
• Symbol digit modalities test
• Verbal fluency (category)
• Reinforcement Learning

**Neuropsychiatric**

*Self-report questionnaires*

• Apathy motivation index
• Barratt impulsivity scale
• Frontal systems behaviour scale
• MOS 36-item short-form health survey
• Obsessive-compulsive inventory
• Pittsburgh sleep quality index
• Speilberger state–trait anxiety index
• Zung self-rating depression scale

**Imaging**

*Volumetric*

• Caudate
• Putamen
• Grey matter
• White matter
• Whole brain

*Diffusion tensor imaging*

• Axial diffusivity
• Fractional anisotropy
• Mean diffusivity
• Radial diffusivity

*NODDI*

• Free water fraction
• Neurite density index
• Orientation dispersion index

*Multiparametric mapping*

• Magnetisation transfer
• Proton density
• R1: longitudinal relaxation rate
• R2*: effective transverse relaxation rate

*Structural connectivity (graph theory)*

• Connection strength
• Efficiency
• Modularity

**Biofluids**

*CSF*

• Mutant huntingtin
• Total huntingtin
• GFAP
• IL-6
• IL-8
• Neurogranin
• NfL
• Total tau
• UCH-L1
• YKL-40

*Plasma*

• GFAP
• NfL
• Total tau

Cambridge Neuropsychological Test Automated Battery (CANTAB)^11^ and Emotion, Motivation, Impulsivity and Social Cognition (EMOTICOM)^12^ are computerised cognitive assessments. CANTAB assesses non-social cognition. EMOTICOM assesses social and emotion cognition. Neuropsychiatric measures were obtained from a series of validated self-report questionnaires. Volumetric imaging provided volume measures adjusted for total intracranial volume. Diffusion tensor imaging,^13^ NODDI,^14^ multiparametric mapping,^15^ and structural connectivity were measured in specified regions of interest. Regions of interest for diffusion tensor imaging and NODDI were the genu, mid-body, and splenium of the corpus callosum, anterior and posterior internal capsule, and external capsule. Multiparametric mapping included the same regions plus caudate and putamen. Connection strength was measured for limbic, executive, and sensorimotor striatum as well as rich club regions (hub regions that have the highest number of connections to other brain regions in the network). Rich club regions included the inferior parietal, superior parietal, precentral, rostral middle frontal, and superior frontal cortices, and the thalamus. Efficiency and modularity are whole brain measures of integration and segregation, respectively. Assays used for biofluid analysis are in the appendix (p 25). Further details for all measures are in the appendix (pp 6–17). MOS=medical outcomes survey. NODDI=neurite orientation dispersion and density imaging. GFAP=glial fibrillary acidic protein. IL=interleukin. NfL=neurofilament light protein. UCH-L1=ubiquitin carboxyl-terminal hydrolase L1. YKL-40 is also known as chitinase 3-like protein 1.

A comprehensive battery of neuropsychological tests, including from the Cambridge Neuropsychological Test Automated Battery (CANTAB)^11^, ^16^, ^17^ and social and emotional cognition and motivation from the Emotion, Motivation, Impulsivity and Social Cognition (EMOTICOM) battery,^12^ was used. Tests were chosen to measure performance across multiple domains for which there was previous evidence of impairment in Huntington's disease, including cognitive flexibility,^18^ planning,^19^ verbal fluency,^20^ emotion recognition,^21^ inhibition,^22^ attention,^22^ learning,^23^ and memory^23^, ^24^ (further details in the appendix, pp 6–10).

A comprehensive battery of neuropsychiatric function was collected using well validated self-report questionnaires, capturing the following domains: depression, anxiety, apathy, sleep, impulsivity, obsessive-compulsive behaviour, frontal behaviour, and general health (panel).

We did neuroimaging for all enrolled participants when possible, but despite initial screening for MRI contraindications, some participants were found to be unsuitable for scanning on the day of their visit and did not undergo MRI scanning. Assessments included volumetric T1-weighted imaging, diffusion-weighted imaging, and novel multiparametric mapping. Volumetric measures of whole brain, striatum (putamen and caudate), grey and white matter, and the ventricles were derived from T1-weighted images; diffusion-weighted imaging data were analysed using diffusion tensor imaging^13^ and neurite orientation and dispersion density imaging (NODDI),^14^ providing measures of white matter microstructure within six prespecified regions of interest (panel), which were selected based on previous work.^25^ Multiparametric mapping provided assessments of myelin and iron within the brain,^15^ which were analysed within the same regions of interest plus caudate and putamen. Structural connectivity metrics were derived from diffusion-weighted imaging for six cortico-striatal measures, 14 cortico-cortical connection measures, and two whole brain network measures, all in right-handed participants only.

We collected biofluids using standardised, well validated conditions, methods, and equipment^26^ (appendix p 17). Total huntingtin (mutant huntingtin and wild type), mutant huntingtin, NfL, YKL-40 (also known as Chitinase-3-like protein), total tau, neurogranin, interleukin-6 (IL-6), interleukin-8 (IL-8), glial fibrillary acidic protein (GFAP), and ubiquitin carboxyl-terminal hydrolase L1 (UCH-L1) were measured in CSF; NfL, total tau, and GFAP were measured in plasma.

### Statistical analysis

The study had 80% power and a 5% risk of type 1 error to reject the primary null hypothesis if, after statistical adjustment for covariates, the group difference was 0·53 within-group standard deviations. This hypothetical difference is consistent with the striatal volume difference between controls and the group furthest from onset in the TRACK-HD study.^4^

Multiple imputation was used to account for missing data (appendix pp 18–19). All measures were processed and analysed blinded to disease status and clinical data. We used general least-squares linear models to assess possible overall group differences and age interactions between groups. Within these same models we controlled and tested possible differences driven by age-by-CAG interaction within the preHD group, since this interaction closely relates to predicted years to onset. Covariates included age, sex, and age interactions with sex. For cognitive measures, we included the national adult reading test score, an estimate of premorbid IQ, and the International Standard Classification of Education, an index of the highest level of education achieved, as covariates. For volumetric imaging measures, total intracranial volume was included as a covariate. Associations between biofluids and cognitive, neuropsychiatric, and imaging measures were investigated. We addressed multiple comparisons via the false discovery rate (FDR), and considered an FDR estimate of less than 0·05 to be significant. Exceptions were the relationship of mutant huntingtin concentrations to age and CAG length—a fundamental a priori hypothesis which was assessed with traditional p values. Biofluid measures deemed exploratory (total huntingtin, GFAP, and UCH-L1) based on the absence of previous published evidence were excluded from FDR correction.

Informed by primary hypothesis results, we did further analyses: a receiver operator characteristic (ROC) curve analysis of YKL-40, CSF, and plasma NfL to assess their ability to distinguish preHD participants from controls; an age-by-NfL concentration comparison combining the HD-YAS and HD-CSF^8^ study cohorts to generate CAG-specific curves across the adult lifespan; and a bootstrapped comparison of caudate and putamen volumes to test for a significant difference in the relationship to gene-carrier status (appendix pp 18–19).

### Role of the funding source

The funder of the study had no role in study design, data collection, data analysis, data interpretation, or writing of the report. The corresponding author had full access to all the data in the study and had final responsibility for the decision to submit for publication.

## Results

We screened 314 individuals for exclusion in this analysis and collected data from 131 of these individuals between Aug 2, 2017, and April 25, 2019; 183 individuals were excluded from the analysis. Common reasons for exclusion included a disease burden score of more than 240 (n=75), contraindications for MRI (n=41), at-risk genetic status (n=23), and significant comorbidity (n=11; four of which were psychiatric). 23 individuals were not included owing to inadequate matching. The final cohort comprised 131 participants (64 preHD and 67 controls), closely matched for age, sex, and education (table). 28 controls were gene negative, 29 were family or partners with no known risk of Huntington's disease, and ten were Huntington's disease community members not at risk of Huntington's disease. The preHD cohort was estimated to be a mean 23·6 years (SD 5·8) from clinical disease onset.

**Table:** Participant demographic characteristics

		**PreHD (n=64)**	**Control (n=67)**	**p value**
Age (years)		29·0 (5·6)	29·1 (5·7)	0·95
Sex				0·81
	Male	30 (47%)	28 (42%)	..
	Female	34 (53%)	39 (58%)	..
Education (years)		16·2 (2·1)	16·3 (2·2)	0·93
NART		102·4 (7·5)	103·5 (8·3)	0·42
UHDRS total motor score*		0 (0–0·25)	0 (0–0)	NA
Total functional capacity†		13 (13–13)	13 (13–13)	NA
CAG repeat length		42·2 (1·6)	NA	NA
Disease burden score		189·3 (39·3)	NA	NA
Estimated years to onset		23·6 (5·8)	NA	NA

Values are means (SD), n (%), or median (IQR). Group comparisons were made using t tests (age, education, and NART) and χ^2^ tests (sex). NA=not applicable. NART=National Adult Reading Test, an estimate for IQ. PreHD=premanifest Huntington's disease. UHDRS=Unified Huntington's Disease Rating Scale.

* Motor scores in both groups were low and within previously reported control ranges,^27^, ^28^ confirming the absence of early Huntington's disease-related motor signs.

† All participants in both groups had a total functional capacity of 13/13, representing no functional impairment, and therefore no p value is provided.

61 (91%) of 67 controls and 62 (97%) of 64 preHD participants were assessed to be suitable for MRI scanning on the day of the procedure and underwent neuroimaging. All but one participant had plasma for analysis; 109 (83%) of 131 participants also underwent optional CSF collection obtained via lumbar puncture (breakdown in appendix, p 24). In the interests of conciseness, we graphically display only selected results: all cognitive and neuropsychiatric results are displayed via radar plots; volumetric imaging results are selected as the most widely used imaging technique in Huntington's disease and as the only imaging domain showing a significant result; biofluid measures that have previously shown differences in preHD are displayed, plus total huntingtin and neurogranin, which are also of substantial interest (total huntingtin is a potential marker of target engagement for huntingtin-lowering therapies and neurogranin is a marker of synaptic function). Details of remaining results are in the appendix (pp 26–38).

**Figure 1:**
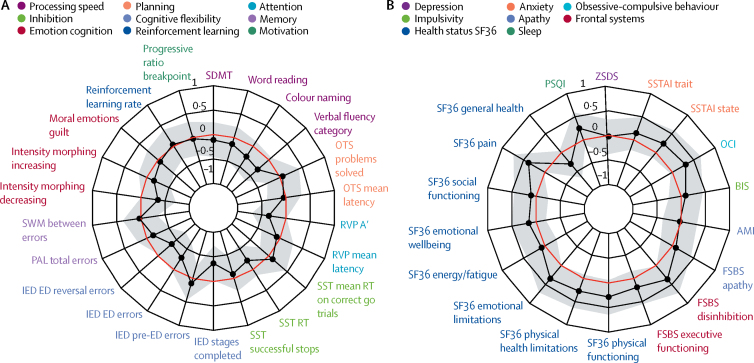
Radar plot showing (A) cognitive and (B) neuropsychiatric variables in the Huntington's disease Young Adult Study The black line shows the standardised mean difference between individuals with premanifest Huntington's Disease (preHD) and controls, with conventional frequentist 95% CI shaded in grey. The red line represents no difference between means (ie, the null hypothesis) and a value within this line represents greater impairment in the preHD group. After false discovery rate correction for multiple comparisons, there were no significant group differences in any cognitive or neuropsychiatric measures. See appendix for further details of these variables (pp 6–12) and discussion (p 22). AMI=apathy motivation index. BIS=Barratt impulsivity scale. ED=extra dimensional. FSBS=frontal systems behavioural scale. IED=intra–extra dimensional set shifting. OCI=obsessive compulsive inventory. OTS=one touch stockings. PAL=paired associates learning. RT=reaction time. RVP=rapid visual processing. RVP A'=a signal detection theory measure of target sensitivity, and mean response latency. SDMT=symbol digit modalities test. SF36=36-item self-report survey. SST=stop signal task. SWM=spatial working memory. SSTAI=Speilberger state trait anxiety inventory. PSQI=Pittsburgh sleep quality index. ZSDS=Zung self-rating depression scale.

There were no significant differences between preHD and controls in any cognitive or neuropsychiatric measure (FDR 0·22–0·87, and 0·31–0·91, respectively; figure 1, appendix pp 26–29). Within the preHD group, there were no significant relationships between cognitive or neuropsychiatric variables and predicted years to onset (FDR 0·12–0·98).

**Figure 2:**
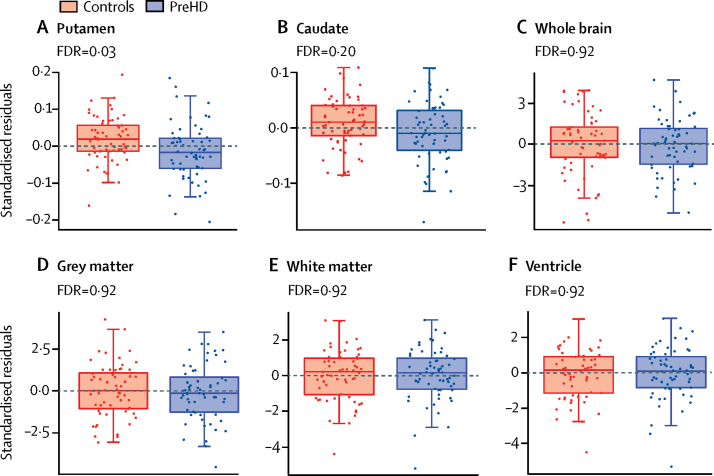
Volumetric MRI Boxplots of standardised residuals (covariate adjusted) for volumes of (A) putamen, (B) caudate, (C) whole brain, (D) grey matter, (E) white matter, and (F) ventricles corrected for intracranial volume. Horizontal lines are the medians, boxes are upper and lower quartiles, and whiskers are 1·5 × IQR. Putamen volume was significantly reduced in individuals with preHD compared with controls (FDR=0·03). None of the other brain measures showed significant between-group differences. FDR=false discovery rate correction. PreHD=premanifest Huntington's disease.

Putamen volumes were significantly smaller in preHD participants compared with controls after FDR correction (FDR=0·03; figure 2). Uncorrected caudate volumes were also smaller in preHD participants (p=0·048), but the corresponding FDR was non-significant (figure 2). Differences were small: the preHD group had 5·5% smaller putamen and 4·0% smaller caudate volumes. Caudate and putamen volume reduction did not show a significant relationship with predicted years to onset in preHD (FDR corrected value 0·54 for both). There were no significant differences between caudate and putamen volume reduction (p=0·30), thus we cannot directly conclude that the putamen showed more disease-related volumetric differences than the caudate. One prominent outlier in the control group had marked NfL elevation for which no additional cause was found (appendix, p 21).

There were no group differences in other brain imaging measures (FDR >0·16). There were no significant group differences in volumes of whole brain, grey or white matter, or ventricles (figure 2; appendix pp 30), nor white matter microstructure as assessed by diffusion tensor imaging and NODDI (FDR 0·27–0·98; appendix pp 31–33). Multiparametric mapping-derived measures of iron and myelin in the striatum and peristriatal white matter were also unchanged in preHD (FDR 0·17–0·99; appendix, pp 34–35), as were the structural connectivity metrics (FDR 0·71–0·99; appendix, pp 36–37). Within the preHD group, there were no significant relationships between any imaging measure and age and CAG length and age-by-CAG interaction (FDR 0·44–0·96).

CSF mutant huntingtin was detectable at low concentrations for all mutation carriers except in three participants, all of whom had a low disease burden score. Higher mutant huntingtin concentrations were associated with increasing CAG length, increasing age, and their interaction (F[3,51]=2·81, p=0·49). CAG and its interaction with age remained significant after controlling for the main effect of age (F[2,51]=3·46, p=0·039). However, 31 (53%) of 58 preHD mutant huntingtin values were between the limit of detection and limit of reliable quantification (8–25 fM), a range in which the output from the assay is not linear. Although this was accounted for in our statistical methods, this interaction should be interpreted with caution. Total huntingtin concentrations were not significantly different between controls and preHD (p=0·23).

**Figure 3:**
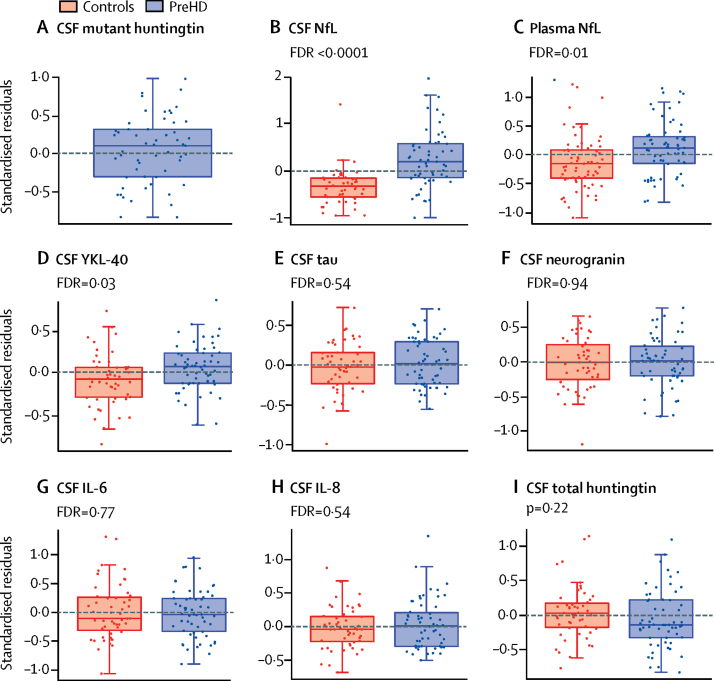
Biofluid measures Boxplots of standardised residuals (covariate adjusted) of (A) CSF mutant huntingtin, (B) CSF NfL, (C) plasma NfL, (D) YKL-40, (E) CSF total tau, (F) CSF neurogranin, (G) CSF IL-6, (H) IL-8, and (I) CSF total huntingtin. Horizontal lines are the medians, boxes are upper and lower quartiles, and whiskers are 1·5 × IQR. All analytes were log transformed. As expected, mutant huntingtin was undetectable in all controls. There were significant differences between individuals with preHD and controls for CSF NfL (FDR<0·0001), plasma NfL (FDR=0·01), and CSF YKL-40 (FDR=0·03). No other analytes showed significant group differences. FDR=false discovery rate correction. NfL=neurofilament light protein. IL=interleukin. PreHD=premanifest Huntington's disease.

The preHD cohort had significantly higher concentrations of CSF NfL, plasma NfL, and CSF YKL-40 than the control cohort (FDR <0·0001, FDR=0·01, and FDR=0·03, respectively; figure 3). CSF NfL concentrations showed a strong association with predicted years to onset in preHD (FDR<0·0001), increasing in those closer to predicted clinical onset. Plasma NfL did not show a significant association with predicted years to onset (FDR=0·18). ROC analysis of CSF NfL, plasma NfL, and CSF YKL-40 gave areas under the curve of 0·79, 0·65, and 0·64, respectively (appendix p 43), implying superior discrimination of CSF NfL over plasma NfL and YKL-40 in individuals far from predicted clinical onset. 31 gene carriers (53%) of 58 had CSF NfL concentrations within the normal control range (95th percentile of controls) and 55 (87%) of 63 had plasma NfL concentrations within normal range. There were no significant differences between the preHD and control cohorts for the other analytes (FDR 0·48–0·94; appendix p 38).

**Figure 4:**
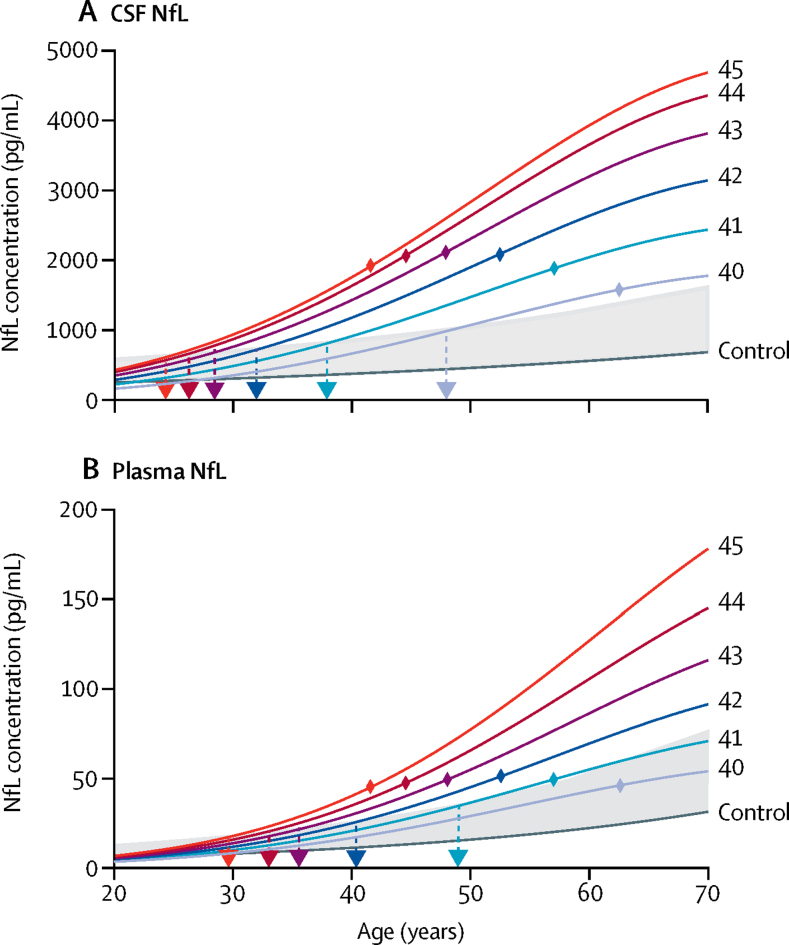
NfL trajectories Associations of NfL concentration in (A) CSF and (B) plasma with age and CAG repeat count from combined datasets of HD-YAS and HD-CSF^8^ (where CAG=Huntington's disease gene carriers' CAG repeat counts). Data were modelled with a polynomial function of age, CAG repeat counts, their squares, and their interactions. NfL concentrations were reverse-transformed from log NfL values. CAG repeat counts are coloured separately and labelled on the right of the image. Shaded in grey is the range between the control curve (dark grey line) and the 95th prediction interval of controls. Dashed arrows show the intercept of NfL trajectory in Huntington's disease and the 95th prediction interval of controls, representing the age at which NfL concentrations become abnormal. Coloured diamonds show the mean age of clinical onset for each CAG based on the Langbehn equation using previously published data.^3^ Further details are in the appendix (p 17). NfL=neurofilament light protein.

By combining our HD-YAS data with baseline values from the HD-CSF study,^8^ we modelled NfL trajectories by CAG count across an age range of 20–70 years (figure 4), showing the age at which NfL is predicted to rise above the 95th percentile of controls for each given CAG count. CSF NfL concentrations increased slowly initially, before accelerating as individuals moved close to predicted clinical onset, followed by a deceleration later in the disease. Plasma NfL showed a similar trajectory, but with concentrations remaining within the normal range for longer. Correlations between biofluids and all other measures are in the appendix (p 21).

## Discussion

**Figure 5:**
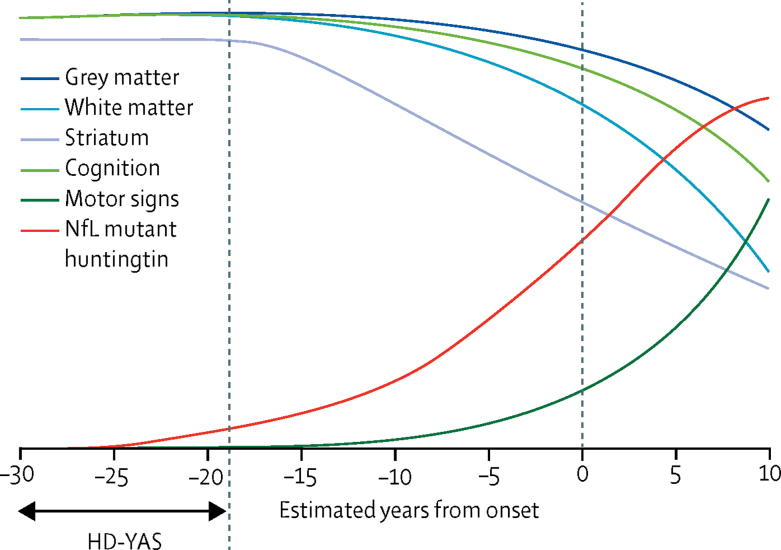
Disease trajectory in Huntington's disease from early adulthood to manifest disease^4^, ^5^, ^8^, ^27^, ^28^, ^29^, ^30^, ^31^, ^32^, ^33^, ^34^, ^35^, ^36^ Evidence-based predictive schematic, with biofluid changes shown in red, brain volumetrics in blue, and functional performance in green. The label HD-YAS shows the range of years to estimated onset represented in our study. NfL and mutant huntingtin are the first pathological changes, occurring at around 24 years before expected symptom onset, with slow increases for approximately 10 years, followed by an acceleration (data from the HD-YAS and the HD-CSF study).^8^ Striatal volumes are slightly smaller than those of age-matched controls at the beginning of adulthood (data from the HD-YAS) and start to decline around 18 years before expected symptom onset. Decline is approximately linear,^29^ and volume reduction is around 50% of control volume by the time of clinical onset.^30^ White matter volume is reduced^4^ and shows higher rates of atrophy^31^, ^32^ than in controls by around 15 years before symptom onset, following a non-linear trajectory.^29^ Grey matter loss extends beyond the striatum later, at around 10 years before symptom onset,^4^, ^33^ after which it progresses non-linearly.^29^ Soft motor signs in the form of increased variability in voluntary movements are apparent by 15 years before symptom onset^4^, ^5^, ^6^, ^27^ and increase non-linearly.^29^, ^34^ Cognitive changes start to emerge approximately 15 years before expected clinical symptom onset,^4^, ^5^, ^35^ declining slowly^31^, ^36^ following a non-linear trajectory.^29^ HD-YAS=Huntington's disease Young Adult Study. NfL=neurofilament light protein.

Results from the HD-YAS, in which we used an extensive clinical testing battery, advanced neuroimaging, and CSF and plasma biofluid analyses suggest that motor, cognitive, and psychiatric function are preserved in gene carriers approximately 24 years from predicted onset of clinical symptoms. There is little evidence of extensive brain atrophy, yet elevated concentrations of CSF NfL are suggestive of subtle neuronal injury in this cohort. We propose that increased CSF NfL and mutant huntingtin concentrations might be the earliest detectable pathological events in Huntington's disease. Combining our novel findings in young adults with multiple large cohorts, we produced an evidence-based predictive schematic of disease trajectory (figure 5), extending the timeline for pathological changes back to the start of adulthood. This model provides crucial new insights into the start of the degenerative process and the evolution of disease markers over time.

Cognitive deficits have been reported previously in premanifest cohorts,^4^ with the PREDICT-HD study suggesting that cognitive function starts to decline around 15 years before clinical onset.^5^ Our cognitive battery represents a comprehensive assessment of cognition in Huntington's disease, including tasks that have not previously been studied in the premanifest stage. Although there were some small group differences in measures of cognitive flexibility, sustained attention, and emotion processing (appendix p 22), none survived multiplicity correction. These differences, driven by individuals closer to disease onset, might herald incipient disruption of executive function. We suggest that measures such as set shifting should be included in future large cohort studies, since they might prove to be the most sensitive cognitive measures in preHD closer to onset. Indeed, in a previous study a task with conceptual overlaps involving set-shifting showed decline after 6 months in preHD participants 16 years from predicted onset.^37^

Increased neuropsychiatric symptoms have also been reported extensively in gene carriers,^4^, ^38^ including in those more than 12 years from predicted onset.^38^ The absence of difference in psychiatric symptoms in this cohort is an important finding, suggesting that Huntington's disease gene carriers do not have appreciable behavioural symptoms simply related to their disease status in early adulthood.

We noted little evidence of abnormality in brain structure at this stage of Huntington's disease. By contrast with previous work in a preHD cohort,^25^ there were no group differences in standard diffusion and NODDI metrics. This finding suggests white matter integrity is maintained at this stage, further supported by the absence of significance in structural connectivity metrics. Previous work has provided indirect evidence of myelin^39^ and iron^40^ disruption in Huntington's disease, but our novel multiparametric mapping did not reveal any regional differences between preHD participants and controls at this stage of the disease process. Brain atrophy has been widely reported in later premanifest cohorts,^5^, ^36^ with caudate atrophy providing the largest effect size of any assessment in the TRACK-HD study.^36^ Both the TRACK-HD^4^ and PREDICT-HD^5^ studies suggested that striatal volumes are reduced compared with controls at least 15 years from expected disease onset. We showed gene carriers to have significantly smaller putamen volumes. However, a statistical comparison of volume reduction between striatal subregions did not provide robust evidence for the putamen being more affected than the caudate. There was little evidence of an association between striatal volume and years to onset or NfL concentrations, suggesting that this might be a neurodevelopmental constitutive difference, which is consistent with previously published work in healthy child and adolescent Huntington's disease gene carriers.^41^ Alternatively, this might be a result of neurodegeneration that is too subtle and variable to show robust associations with disease burden at this stage; longitudinal follow up might help to resolve this question. Nevertheless, the small effect size and absence of association with disease burden indicates striatal atrophy might have little use as a marker of progression at this stage of Huntington's disease.

Even in this far-from-onset cohort, NfL concentrations were significantly increased. NfL concentrations have previously been shown to be closely associated with brain volumes, clinical scores, and subsequent clinical onset and progression in Huntington's disease.^7^, ^8^ CSF NfL had the highest effect size of any measure in this study and was the only measure showing a significant increasing association with estimated years to onset. Through modelling NfL with age, we showed the approximate age at which NfL becomes abnormal for a given CAG length. However, 31 preHD participants (53%) had CSF NfL concentrations within the 95th percentile of controls, suggesting we have identified a crucial point at which CSF NfL begins to rise. Our ROC analysis suggested that plasma NfL is less sensitive for detecting early neurodegeneration in this cohort, with 55 preHD values (87%) within the 95th control percentile; this is in contrast to the near-equivalence of CSF and plasma NfL in manifest Huntington's disease.

NfL is therefore a potential candidate to provide a measure of disease progression in early preHD and might eventually be used as a marker of response to treatment in future preventive trials. Future trials targeting far-from-onset individuals might enrich recruitment by using age and CAG length (eg, using the CAG-age-product score) and NfL above a pre-defined cutoff to increase the likelihood of seeing measurable change over a typical trial timeframe. The use of NfL as an enrichment marker requires further validation, and there are still uncertainties regarding the use of NfL as a marker of response to treatment. Early data from an antisense oligonucleotide trial^2^ reported transiently increased CSF NfL in response to huntingtin-lowering treatment. We will develop a better understanding of NfL's response to therapeutic interventions as its performance in numerous clinical trial programmes becomes apparent, alongside the performance of each therapy. Although non-specific to Huntington's disease, the usefulness of NfL is strengthened in this case since individuals with incipient mutant huntingtin neuropathology can be reliably identified by genetic testing, and other neurodegenerative and neurovascular diseases are rare in the age ranges studied in this cohort. Despite its practical and cost advantages, plasma NfL might not be as sensitive as CSF NfL in early preHD.

A key aim of current therapies in development for Huntington's disease is to reduce mutant huntingtin in the nervous system, which has already been successfully used as a marker of target engagement for current trials of huntingtin-lowering therapies in manifest Huntington's disease.^2^ In our cohort, further from predicted onset, mutant huntingtin concentrations were lower than previous reports.^8^, ^26^ Only 23 gene carriers (40%) had concentrations above the limit of reliable quantification. This finding suggests that, although suppression of mutant huntingtin could be a viable measure of target engagement for clinical trials at this early stage, it would be unable to quantify the percentage of huntingtin-lowering to assess dose-response. The presence of the mutant *HTT* gene did not have any effect on total huntingtin concentrations; total huntingtin concentrations in CSF have not, to our knowledge, been examined in controls and Huntington's disease mutation carriers before. With concentrations consistently above the limit of quantification, this measure might provide a more reliably quantified marker of target engagement in total huntingtin-lowering therapies than mutant huntingtin at this early stage.

Our finding of increased concentrations of YKL-40 in preHD and their close association with CSF mutant huntingtin and NfL suggests astrocytic activation, due to mutant huntingtin-induced neuronal injury or cell-autonomous effects of mutant huntingtin astrocytes.^42^ ROC analysis suggested that YKL-40 is less sensitive or specific than plasma and CSF NfL in early preHD (appendix p 43). Tau,^43^ IL-6, and IL-8,^44^ which were previously reported to be increased in preHD, were not significantly different in this preHD cohort further from predicted clinical onset (appendix p 23).

Our study has implications not only for our understanding of Huntington's disease processes, but also in identifying the optimum time for treatment interventions. Ideally in the future, effective treatments will be administered before widespread neuronal damage, but there is likely to be a complex trade-off between the benefits of slowing the disease at that point and any negative effects of long-term treatment. This risk-benefit decision will also vary depending on the nature of the treatment and the circumstances of the gene carrier.

With respect to limitations, our study was powered to detect plausible disease-related changes in striatal volumes, and it might have been underpowered to detect associations with age and CAG repeat length. Given our sample size, our negative findings suggest an absence of substantial differences in these measures between the two groups. This finding does not rule out the possibility of subtle early preHD changes, but does show that any such differences would be difficult to separate from natural variation among young adults. The measures used in this study, although comprehensive, were not exhaustive and other measures might have shown sensitivity in preHD. However, we maximised our sensitivity by selecting measures that were most likely to show early disease-related changes. The cognitive and neuropsychiatric batteries were selected based on previous work^4^, ^45^ suggesting that these domains were most likely to show early deficits, as well as including novel assessments in CANTAB and EMOTICOM batteries shown to be highly sensitive in young cohorts. We also used state-of-the-art high-resolution 3T imaging with validated analysis methods to maximise our sensitivity to disease-related brain changes. The increasing availability of 7T imaging might provide yet more detailed evidence on the underlying Huntington's disease pathology in the future. Longitudinal follow up of this cohort will be important to address these issues, while also providing further clarity on the biomarkers that are most suitable for clinical trials in such far-from-onset cohorts.

In summary, by identifying a cohort of preHD individuals with no detectable functional impairment but who begin to exhibit subtle elevations in select biological measures of neurodegeneration, we have highlighted a crucial point early in the disease process. Intervening at this stage might offer the prospect of delaying or preventing further neurodegeneration while function is intact, giving gene carriers many more years of life without impairment.
