# Supplementary material for: Biological and clinical characteristics of gene carriers far from predicted onset in the Huntington's disease Young Adult Study (HD-YAS): a cross-sectional analysis
Source: Lancet Neurol. 2020 Jun;19(6):502–12. doi: 10.1016/S1474-4422(20)30143-5 (PMC7254065; doi:10.1016/S1474-4422(20)30143-5)
Supplement: Supplementary appendix [file mmc1.pdf]

THE LANCET
Neurology

Supplementary appendix

This appendix formed part of the original submission and has been peer reviewed. We post it as supplied by the authors.

Supplement to: Scahill RI, Zeun P, Osborne-Crowley K, et al. Biological and clinical characteristics of gene carriers far from predicted onset in the Huntington's disease Young Adult Study (HD-YAS): a cross-sectional analysis. *Lancet Neurol* 2020; **19:** 502–12.

Supplementary Material

Contents

Methods....................4

Participants....................4

Recruitment....................4

Eligibility screening....................4

Cognition....................6

CANTAB Intra-Extra Dimensional Set Shifting (IED)....................7

CANTAB One-Touch Stockings of Cambridge (OTS)....................7

CANTAB Rapid Visual Information Processing (RVP)....................7

CANTAB Stop Signal Test (SST)....................7

CANTAB Paired Associates Learning (PAL)....................8

CANTAB Spatial Working Memory (SWM)....................8

EMOTICOM Emotional Intensity Face Morphing....................8

EMOTICOM Moral Emotions Test....................9

EMOTICOM Progressive Ratio....................9

Semantic Verbal Fluency....................9

Stroop Word Reading Test....................9

Stroop Colour Naming Test....................9

Symbol Digit Modalities Test (SDMT)....................10

Reinforcement Learning....................10

Neuropsychiatry....................10

Zung Self-Rating Depression Scale (SDS)....................11

Spielberger State/Trait Anxiety (STAI) 11
Barratt Impulsivity Scale (BIS-11) 11
Frontal Systems Behavioural Scale (FrSBE) 11
Obsessive-Compulsive Inventory (OCI-R) 11
Apathy Motivation Index (AMI) 11
Pittsburgh Sleep Quality Index (PSQI) 12
MOS 36-Item Short-Form Health Survey (SF-36) 12
Imaging 12
Image Acquisition 12
Volumetric MRI 13
Diffusion imaging 13
Multiparametric mapping 14
Structural Connectivity 15
Biofluids 17
Statistics 17
Statistical software 19
Results 19
Research genotyping 19
Cognition 19
Neuropsychiatry 20
Imaging 20
Biofluids 21
mHTT and NfL predictions of other Huntington’s disease measures 21
Discussion 22
Cognition 22
Imaging 23

Biofluids 23

Supplementary Table 1: Number of assessments by modality. 24

Supplementary Table 2: Biofluid assay details 25

Supplementary Table 3: Cognitive Results 26

Supplementary Table 4: Neuropsychiatric Results 28

Supplementary Table 5: Volumetric Results (corrected for intracranial volume) 30

Supplementary Table 6: Diffusion Results 31

Supplementary Table 7: MPM Results 34

Supplementary Table 8: Structural Connectivity Results 36

Supplementary Table 9: Biofluid Results 38

Supplementary Figure 1: Overview of the HD Young Adult study assessments within each domain. 39

Supplementary Figure 2: Composite image of the CANTAB tests 40

Supplementary Figure 3: Composite image of the EMOTICOM tests 41

Supplementary Figure 4: Summary of connectivity processing pipeline 42

Supplementary Figure 5: ROC curves for NfL and YKL-40 43

References 45

Methods

Participants

Recruitment

Participants were identified and recruited from the Enroll-HD study https://www.enroll-hd.org/, regional Genetic and Huntington's disease centres across the UK who were established as patient identification sites, and through broader efforts such as via the Huntington's Disease Association https://www.hda.org.uk/ and the Huntington's Disease Youth Organisation https://hdyo.org/. We did not perform 1:1 matching but instead matched our groups as closely as possible for age, gender and education on overall mean and variance. This was done as an ongoing process, regularly monitoring means and standard deviations of the two populations as recruitment progressed and where necessary targeting specific demographics to ensure close matching.

Eligibility screening

Prior to enrolment, each participant was interviewed to determine whether they met the eligibility criteria below. For gene carriers, CAG expansion in the Huntington's disease gene was confirmed by obtaining the genetic report from an accredited laboratory.

The first participant was enrolled in August, 2017, and all assessments were completed by April, 2019 at the National Hospital for Neurology and Neurosurgery, UK.

Inclusion criteria

a. Are 18-40 years of age, inclusive; and

b. Are capable of providing informed consent and

c. Are capable of complying with study procedures and

For the **Healthy Control** group, participants eligible are persons who meet the following criteria:

d. Have no known family history of Huntington's disease (family control or community control)*; or

e. Have known family history of Huntington's disease but have been tested for the huntingtin gene CAG expansion and are not at genetic risk for Huntington's disease (CAG < 36) (gene negative).

For the **Young Adult Premanifest Huntington's disease** group, participants eligible are persons who meet the additional following criteria:

f. Do not have clinical diagnostic motor features of Huntington's disease, defined as Unified Huntington's Disease Rating Scale (UHDRS) Diagnostic Confidence Score[1] < 4; and

g. Have CAG expansion ≥ 40 and

h. A disease burden score (DBS) ≤ 240[2] **

* Family controls were partners or spouses of someone either with the Huntington's disease gene or at risk of Huntington's disease due to having a 1st degree relative with Huntington's disease. Community controls were either friends of someone with or at risk of Huntington's disease, or from the wider Huntington's disease community recruited via advertisement through Huntington's disease support groups.

** The rationale for this DBS cut-off is that this boundary corresponds approximately to >18 years to estimated disease onset according to the Langbehn formula[3].

Exclusion criteria

a. Current use of investigational drugs or participation in a clinical drug trial within 30 days prior to study visit; or

b. Current intoxication, drug or alcohol abuse or dependence; or

c. If using any antidepressant, psychoactive, psychotropic or other medications or nutraceuticals used to treat Huntington's disease, the use of inappropriate (e.g., non-therapeutically high) or unstable dose within 30 days prior to study visit; or

d. Significant medical, neurological or psychiatric co-morbidity likely***, in the judgment of the Principal Investigator, to impair participant's ability to complete essential study procedures; or

e. Predictable non-compliance as assessed by the Principal Investigator; or

f. Inability or unwillingness to undertake any of the essential study procedures; or g. Needle phobia; or

h. Contraindication to MRI, including, but not limited to, MR-incompatible pacemakers, recent metallic implants, foreign body in the eye or other indications, as assessed by a standard pre-MRI questionnaire; or

i. Pregnant (as confirmed by urine pregnancy test); or

j. Claustrophobia, or any other condition that would make the subject incapable of undergoing an MRI.

For the optional cerebrospinal fluid (CSF) collection only

k. Needle phobia, frequent headache, significant lower spinal deformity or major surgery; or

l. Antiplatelet or anticoagulant therapy within the 14 days prior to sampling visit, including but not limited to: aspirin, clopidogrel, dipyridamole, warfarin, dabigatran, rivaroxaban and apixaban; or

m. Clotting or bruising disorder; or

n. Screening blood test results outside the clinical laboratory's normal range for the following: white cell count, neutrophil count, lymphocyte count, haemoglobin (Hb), platelets, prothrombin time or activated partial thromboplastin time; or

o. Screening blood test results for C-reactive protein >2× upper limit of normal; or

p. Exclusion during history or physical examination, final decision to be made by the Principal Investigator; including but not limited to:

i any reason to suspect abnormal bleeding tendency, e.g. easy bruising, petechial rash; or ii any reason to suspect new focal neurological lesion, e.g. new headache, optic disc swelling, asymmetric focal long tract signs; or iii any other reason that, in the clinical judgment of the operator or the Principal Investigator, it is felt that lumbar puncture is unsafe.

*** Comorbidities are assessed for during an interview asking about current and previous medical and drug history. The T1 weighted MRI brain was reviewed by an experienced consultant neuroradiologist and CSF white and red cell counts were also reviewed to further ensure absence of neurological comorbidity.

An overview of all assessments performed for each modality is provided in Supplementary Figure 1.

Cognition

Rationale for the Test Battery

The battery covers eight domains previously shown to be important in Huntington's disease, with the addition of social cognition, which has not yet been widely studied. It contains tests previously shown to be sensitive to pre-manifest Huntington's disease such as the Cambridge Neuropsychological Test Automated Battery (CANTAB) Intra-Extra Dimensional Set Shifting (IED) test and semantic verbal fluency[4]. However, we have not included more detailed tests of language function. To our knowledge, we have not omitted any tests with demonstrable sensitivity to far from onset, premanifest Huntington's disease.

CANTAB Intra-Extra Dimensional Set Shifting (IED)

The IED (Supplementary Figure 2A) is a 7-minute test measuring cognitive flexibility and has similarities to a computerised version of the Wisconsin Card Sorting test. It initially features rule acquisition and reversal and then attentional set formation and set shifting. Participants are presented with two artificial dimensions including pink shapes and white lines. Through trial and error the participant must select the correct rule. After six correct responses, the stimuli and/or rule changes. There are 9 stages to the test. Initially the test presents simple stimuli with just one dimension (pink shapes). These later change to compound stimuli (white lines overlaid on pink shapes). Early in the test the shifts are intra-dimensional (ID) (pink shapes are relevant) to establish set formation. Then at stage 8 a crucial extra-dimensional (ED) shift (white lines become relevant) occurs (attentional set-shifting). This latter stage is followed by a final reversal of the rule. Outcomes measures include the number of pre-ED errors, ED shift errors, ED reversal errors and stages completed.

CANTAB One-Touch Stockings of Cambridge (OTS)

The OTS is a modified test of visuospatial planning and working memory based on the Tower of London which takes about 10 minutes to complete. Participants are shown example configurations of three coloured balls (Supplementary Figure 2B). There are two displays and participants are asked the number of moves required to match their display to the example display without actually moving the balls. The problems differ in the number of moves required to match the example configuration, starting at one move progressing to six moves. The outcome measures include average response latency and number of problems solved efficiently at the first choice.

CANTAB Rapid Visual Information Processing (RVP)

The RVP is a 10 minute test which measures sustained attention by presenting a rapid stream of digits and requiring participants to detect target sequences. A white box is displayed in the centre of the screen in which digits 2-9 are rapidly presented at 100 digits per minute (Supplementary Figure 2C). Participants are required to detect target sequences (e.g. 2-4-7, 3-5-7 or 4-6-8) and respond to this target sequence as quickly as possible. Outcome measures include A', a signal detection theory measure of target sensitivity, and mean response latency.

CANTAB Stop Signal Test (SST)

The SST is a test of response inhibition (impulse control) and takes 20 minutes to complete. The participant is shown an arrow in the centre of the screen and must respond with a button depending on the direction the arrow

is pointing (left or right); (Supplementary Figure 2D). If an audio tone is presented together with the arrow, the participant must withhold making the response (inhibition). The outcome measures are stop signal reaction time (SSRT), mean reaction time on go trials and the proportion of successful stops. The ‘last-half’ default setting was used, which calculates the SSRT from the last half of the trials.

CANTAB Paired Associates Learning (PAL)

The CANTAB PAL (Supplementary Figure 2E) is an eight-minute test assessing visuospatial memory and learning. Boxes are displayed on the screen in a spatial array and opened in a random order. One or more boxes contain a visual pattern. The patterns are subsequently displayed one by one in the middle of the screen and the participant must select the box in which the pattern was previously presented. If the participant makes an error, the boxes are opened in the same order again. This is to remind the participant of the locations of the patterns before they attempt to remember again. The outcome measure is the total number of errors adjusted (errors added for stages not completed).

CANTAB Spatial Working Memory (SWM)

The CANTAB SWM (Supplementary Figure 2F) is a nine-minute test assessing spatial working memory. Test performance requires the retention and manipulation of visuospatial information. Coloured boxes are shown on screen and participants must select a box with a token. The token is stored on the edge of the screen and will not appear in the same location for the rest of the trial. Therefore, returning to the same location on the next search is an error. The colour and position of the boxes used are changed from trial to trial to discourage the use of stereotyped search strategies. The outcome measure is the total number of between search errors.

The EMOTICOM battery was recently designed to measure aspects of social and emotional cognition and motivation[5]. We employed three subtests from the battery designed to assess emotion processing, motivation and social cognition.

EMOTICOM Emotional Intensity Face Morphing

The intensity morphing test (Supplementary Figure 2A) measures the emotional intensity at which participants recognise a facial emotion. In a 10 minute test, participants are presented with faces that either increase or decrease in emotional intensity. Participants are required to respond when they either first see the emotion (increasing) or can no longer see the emotion (decreasing). The outcome measures were average detection threshold of sad faces, for the increasing and decreasing condition separately.

EMOTICOM Moral Emotions Test

In the 20 minute moral emotions test (Supplementary Figure 2B) participants view cartoon figures depicting moral scenarios. Participants are asked to rate their levels of guilt, shame, annoyance and feeling "bad" following each of the cartoons. Half of the cartoons are portrayed as deliberate harm and half as unintended harm. Moreover, the participants were asked to rate their emotions from the perspective of both the victim and the perpetrator. The main outcome measure defined for this study was the guilt score collapsed across all conditions (deliberate vs unintentional and perpetrator vs victim).

EMOTICOM Progressive Ratio

The progressive ratio test (Supplementary Figure 2C) is a measure of motivation and effort and is adapted from similar animal tests[5,6]. Participants are shown four red squares and are asked to select the odd one out (i.e., the large square). Participants are rewarded progressively less per trial. Initially participants receive £1 for four presses, this then doubles to 8, 16 and 32 presses before another reward. The incentive is then decreased to 20p and requires 4, 8, 16, 32 and 64 presses for a reward. On the final block participants are only rewarded with 4p and must respond 4, 8, 16, 32, 64 and 128 times for a reward. The total number of trials is 436. Participants are told they may end the test at any point, but they must stay and face the screen for the remaining time (for 20 minutes minus the time they performed the test). The outcome measure is the breakpoint, which is calculated as the maximum total number of trials performed before the participant no longer wishes to continue the test.

Semantic Verbal Fluency

The verbal fluency test is a short test of semantic verbal retrieval. Participants are asked to list as many items as possible in a particular category (animals) in 60 seconds. The outcome measure is the number of words recited correctly.

Stroop Word Reading Test

During the word reading test, participants are asked to read the words (colours) on the page as quickly possible. The outcome measure was the number of words read within 45 seconds.

Stroop Colour Naming Test

During the colour naming test, participants are asked to name the colours on the page as quickly as possible. The outcome measure was the number of colours named within 45 seconds.

Symbol Digit Modalities Test (SDMT)

The SDMT is a short test, of psychomotor speed. It involves a substitution task, whereby using a reference key, the participant has 90 seconds to pair specific numbers and geometric figures.

Reinforcement Learning

Participants completed a reinforcement learning task in which the aim was to maximise rewards by learning to choose the best symbol from a pair of abstract symbols displayed on the screen[7,8]. One of the symbols was associated with a "better" outcome with a probability of 0.8 and the other symbol was associated with the same outcome with a probability of only 0.2. Participants saw three pairs of such symbols corresponding to the three conditions – gains, neutral and loss. With the gain frame the better outcome was to win fictional money (£1) as compared to earning no reward. In the loss frame, participants could either receive no reward or lose £1. In the neutral conditions, both outcomes yielded no reward. In the gain condition if participants won, they saw a high resolution image of a £1-coin with a green surrounding halo. If they lost, they saw the same £1-coin image with a red cross superimposed over it indicating they had lost money. In both gain and loss conditions the alternative outcome was to see the word 'Nothing' appear on the screen. In the neutral condition the two outcomes were either an empty grey disc the same size as the pound coin or the word 'Nothing'. Stimuli were placed above and below a central fixation cross. To choose the upper stimulus participants were required to make a 'Go' response and press a button on an fMRI compatible button box using their right hand. Alternatively, to choose the lower symbol participants withheld response for 3 seconds. Symbol position was random in each trial, so position and value were orthogonal. After either a button press or 3 second delay period, choice was displayed for a jittered interval ranging from 0.5 – 2 seconds drawn from an exponential distribution. Outcome was then displayed for 3 seconds followed by a jittered inter-trial interval (ITI) ranging from 2-6 seconds drawn from an exponential distribution. Participants were shown each pair of stimuli 30 times with a total of 90 choices per run. Compared to previous research, is important to note that participants were not paid for study participation or for performance. The outcome measure was total percentage correct over both gains and losses.

Neuropsychiatry

To evaluate the prevalence of neuropsychiatric symptoms, the following additional assessments were administered:

Zung Self-Rating Depression Scale (SDS)

The SDS[9] is a self-rated scale that is a well validated screening tool for depression. It covers affective, psychological and somatic symptoms associated with depression. Items are scored on a four-point scale.

Spielberger State/Trait Anxiety (STAI)

The STAI[10] is a well validated and commonly used self-report measure of anxiety, providing assessment of both state and trait levels of anxiety. Items are scored on a four-point scale that reflects frequency of anxious thoughts/behaviours.

Barratt Impulsivity Scale (BIS-11)

The BIS[11] is a self-report questionnaire designed to assess the personality/behavioural construct of impulsiveness. It measures three factors, including attentional, motor and non-planning impulsivity. Items are scored on a four-point scale relating to frequency of behaviours.

Frontal Systems Behavioural Scale (FrSBE)

The FrSBE[12] provides a brief but reliable and valid measure of three frontal systems behavioural syndromes: 1) apathy; 2) disinhibition; and 3) executive dysfunction. The FrBSE is a 46-item behaviour rating scale. It includes a Total Score and scores across the three subscales, where 14 items relate to apathy, 15 items to disinhibition and 17 items to executive dysfunction.

Obsessive-Compulsive Inventory (OCI-R)

The OCI[13] is a brief self-report instrument to determine severity of obsessive and compulsive behaviours. The items are scored on a five-point scale identifying how often an individual is distressed by behaviours relating to washing, checking, ordering, obsessing, hoarding and neutralising.

Apathy Motivation Index (AMI)

Apathy is a disorder of motivation characterised by reduced action initiation and goal-directed behaviour. The AMI[14] is a recently developed (adapted from the Lille Apathy Rating Scale[15]) apathy scale assessing three distinct subtypes of apathy: 1) behavioural activation; 2) social motivation; and 3) emotional sensitivity. The items are assessed on a five-point scale that represents how true each statement is.

Pittsburgh Sleep Quality Index (PSQI)

The PSQI[16] is a self-report questionnaire assessing several sub-categories including, subjective quality of sleep, sleep onset latency, sleep duration, sleep efficiency, presence of sleep disturbances, use of hypnotic-sedative medication and presence of daytime sleepiness.

MOS 36-Item Short-Form Health Survey (SF-36)

The SF-36[17] is a self-report scale assessing eight health concepts: 1) limitations in physical activities because of health problems; 2) limitations in social activities because of physical/emotional problems; 3) limitations in usual role because of physical health problems; 4) bodily pain; 5) general mental health (psychological distress and well-being); 6) limitations in usual role because of emotional problems; 7) vitality (energy and fatigue); and 8) general health.

Imaging

Image Acquisition

Imaging was acquired on 123 participants (62 preHD and 61 controls), with the remaining 8 study participants excluded due to claustrophobia or contraindications to scanning identified after screening. All MRI data were acquired on a 3Tesla Prisma scanner (Siemens Healthcare, Germany) with radiofrequency body coil for transmission and a 64-channel head coil for signal reception using a protocol optimised for this study. The T1weighted (T1w) images were acquired using a 3D Magnetization Prepared Rapid Gradient Echo sequence with the following parameters: repetition time (TR) =2530ms; time to echo (TE) =3.34ms; inversion time = 1100ms; flip angle = 7º; field of view=256x256x176mm^3 and a resolution of 1.0x1.0x1.0 mm^3. Diffusion weighted images (DWI) were acquired using a multiband spin-echo echo planar imaging (EPI) sequence with time-shifted RF pulses[18] with acceleration factor 2, TR=3260ms, TE=58ms, flip angle=88º, field of view=220x220mm^2, with 72 slices collected at a resolution of 2x2x2mm^3. The multi-shell data consisted of b-values of 0 (n=10), 100 (n=8), 300 (n=8), 1000 (n=64) and 2000 (n=64) s/mm^2.

The Multiparametric mapping (MPM) acquisition protocol consisted of three differently weighted 3D multiecho Fast Low Angle Shot acquisitions: Magnetisation Transfer weighted (MTw), Proton Density weighted (PDw) and T1w in addition to two scans collected to estimate participant-specific field inhomogeneities. The MTw, PDw and T1w scans were all acquired using a field of view of 256x224x179 mm^3, TR=25ms, flip angle of 6º and resolution of 0.8x0.8x0.8mm^3. To improve image quality, i.e. maximize signal to noise ratio and minimize geometric distortion, eight gradient echoes from 2.34-18.44ms were acquired for the PDw and T1w images and

six from 2.34 – 13.84ms for the MTw image. B1 Transmit bias field maps were collected using a 3D Echo Planar Imaging acquisition of spin-echo and stimulated echo images. 48 slices, TR=500ms, TE1=39.06, TE2=130ms, slice thickness=4 mm; field-of-view: 256 × 192 × 192 mm^3. Finally, the field maps were acquired with 64 slices using TR=1020ms, TE1=10ms TE2=12.46ms, slice thickness=4 mm; field-of-view: 192x192 mm^3. Parallel imaging acceleration was used (GRAPPA) and 3D distortion correction was applied to all images..

Volumetric MRI

T1w images were bias corrected using N3 bias correction[19] within the Medical Imaging Display and Analysis System (MIDAS)[20] software tool. Whole brain, total intracranial and ventricular volumes were delineated using MIDAS with previously described semi-automated protocols[21,22]. Putamen and caudate volumes were derived using the Multi Atlas Label Propagation with Expectation Maximisation-Based Refinement (MALP-EM) software[23] (version 1.2; https://github.com/ledigchr/MALPEM), after linear registration into standard space using the International Consortium of Brain Mapping 152 template (http://www.bic.mni.mcgill.ca/ServicesAtlases/ICBM152NLin2009). Voxel-based morphometry[24] (Statistical Parametric Mapping version 12; https://www.fil.ion.ucl.ac.uk/spm/software/spm12/) was used with the Computational Anatomy Toolbox (version 12; http://www.neuro.uni-jena.de/cat12/CAT12-Manual.pdf) to generate grey and white matter volumes in native space. All segmentations were visually inspected blind to disease status. No segmentations failed this quality control.

Diffusion imaging

DWI images were corrected for susceptibility-induced artefacts using *topup*, and for motion and eddy-current induced artefacts using *eddy*[25–27], both from FMRIB Software Library (FSL) (version 5.0.11; https://fsl.fmrib.ox.ac.uk/fsl/fslwiki).

Diffusion tensors (DT) were fitted to the artefact-corrected DWI data using FSL *dtifit*. DT indices, including fractional anisotropy (FA), mean diffusivity (MD), axial diffusivity (AD) and radial diffusivity (RD), were then calculated using DTI-TK (version 2.3.3; http://dti-tk.sf.net).

The Neurite Orientation and Dispersion Density (NODDI) model[28] was fitted to artefact-corrected DWI data using the Accelerated Microstructure Imaging via Convex Optimization toolbox (version 10a65b0, Nov 2016; http://amico.sourceforge.net/)[29]. NODDI distinguishes between markers of axonal density and axonal spatial

organization, while removing the potentially confounding effect of free water. Output indices were neurite density index (NDI), orientation dispersion index (ODI) and free water fraction (FWF).

The average values of DT and NODDI indices were determined for six pre-specified white matter regions of interest (ROIs) in each participant, as described previously[30]. The ROIs are genu, splenium and mid-body of the corpus callosum, anterior and posterior limbs of the internal capsules, and the external capsules. For the internal and external capsules, the left and right hemispheric regions were combined as one. The spatial definition of the ROIs was extracted from the Johns Hopkins University (JHU) white matter atlas[31]. The delineation of the ROIs in individual subjects was accomplished by spatially aligning each participant diffusion data with the JHU white matter atlas. The alignment was achieved via a bootstrapped population template using linear and non-linear deformation in DTI-TK, a state-of-the-art white matter-specific registration algorithm[32,33].

Multiparametric mapping

MPM data were collected on 121 participants (1 preHD and 1 control who were scanned did not undergo MPM imaging due to claustrophobia since this was the last sequence of the acquisition). The MPM multi-echo protocol was created to estimate the longitudinal relaxation rate (R1), the effective transverse relaxation rate (R2*) the proton density (PD) and magnetization transfer (MT)[34]. Data collection involved the acquisition of at least six images at different TE lengths for each of the PD, T1 and MT weighted acquisitions. These images were pre-processed to generate PD, R1, MT and R2* quantitative maps (as described in[34]). Additional B1 and Field Maps were collected to correct for field inhomogeneities. R1 represents estimates of iron and myelin, R2* iron, MT myelin and PD sensitivity to water content. Two MPM datasets (1 control, 1 preHD) were excluded due to motion, three for processing failure (all preHD) and three for motion on the R2* scans only (all controls).

Scans were first converted to NIfTI format, and visual quality control performed. Pre-processing was then performed using default settings within the histology using MRI (hMRI) toolbox version 0.2.0[35], within the Statistical Parametric Mapping software (SPM version 12) in matlab version R2012b (The Mathworks Inc, Natick, MA, USA). RF sensitivity bias correction calculated via Unified Segmentation, and B1 sensitivity bias correction calculated via the RF transmit (B+1) and receive (B−1) field measurements. Quantitative maps were then calculated from the three multi-echo spoiled gradient echo scans; these were visually examined after preprocessing.

Eight regions of interest were specified *a priori* .The caudate and putamen regions generated by MALP-EM were registered and resampled to native MPM space for each participant via an affine registration using NiftyReg software[36]. These regions were then binarized and eroded by one voxel in all planes to ensure that they

contained no CSF or white matter. Identical to the diffusion processing above, white matter regions from the JHU White Matter Label Atlas[31] were used to generate values for the external capsule, posterior and anterior internal capsule, posterior- mid- and anterior- corpus callosum. These were registered and resampled to native MPM space for each participant via affine transformation, followed by non-linear registrations[36]. All masks were overlaid on the MPM maps and visually checked to ensure successful registration. Quantitative values representing the average value within each region were then extracted for each quantitative map, for every participant. Quantitative values for the control group were found to be in line with previously published values[36,37].

Structural Connectivity

Structural connectivity analysis was performed for 107 participants. Only right-handed participants were included to avoid confounding effects caused by differences in structural connectivity in those who are right hemisphere dominant.

Processing for the structural connectivity is summarised in Supplementary Figure 3. Seventy-six cortical ROIs were segmented on the T1w images using FreeSurfer (version 6.0.0; https://surfer.nmr.mgh.harvard.edu/)[38]. The amygdala was not included as automatic segmentation of this structure is not sufficiently reliable[39]. The cerebellum was not included as diffusion data was incomplete. Tissue partial volume maps of the brain white matter, grey matter, and CSF were prepared for anatomically constrained tractography (ACT)[40].

Previous studies have demonstrated that cortical rich club (hub regions that have the highest number of connections to other brain regions in the network) and striatal structural connections are selectively vulnerable in gene carriers closer to expected disease onset, with an accompanying loss of integration and increased segregation across the brain network[41,42]. We selected *a priori* 12 cortical rich club regions and 6 striatal regions to investigate whether gene carriers approximately 24 years from predicted onset show subtle early differences in structural connectivity in these selectively vulnerable regions.

The striatum was segmented using an atlas that splits the structure into 3 sub-regions in each hemisphere according to cortical-striatal anatomical connections[43]. These subregions are labelled limbic, executive and sensorimotor based on the dominant cortical connectivity to each striatal subregion.

A surface-based registration was used to register the atlas to participant T1 space before registering to participant diffusion space using the NiftyReg toolkit[36]. All diffusion processing steps were conducted using

commands either implemented within MRtrix3 (version 3.0; www.mtrix.org), or using MRtrix scripts that interfaced with external software packages.

Preprocessing of diffusion-weighted images included eddy-current correction and motion correction[26] and bias field correction[44]. Following these steps, fibre orientation distributions (FODs) were computed using multi-shell, multi-tissue constrained spherical deconvolution with group averaged response functions for white matter, grey matter and CSF[45]. Multi-tissue informed log-domain global intensity normalisation was then performed.

Whole-brain probabilistic tractography was performed in participant-space using the 2nd order integration over fibre orientation distributions (iFOD2) algorithm[46] with a FOD threshold of 0.06. Ten million streamlines were generated for each scan using dynamic seeding in the white matter. The 'back-tracking' mechanism was used within the ACT framework[40] to allow tracks to be truncated and re-tracked if poor structural termination was encountered.

Connectomes were constructed by combing streamline tractograms with the participant's grey matter parcellation. Streamlines were assigned to the closest node within a 2-mm radius of each streamline endpoint[47]. Structural connections were weighted by streamline count and a cross-sectional area multiplier, as implemented in Spherical-Deconvolution Informed Filtering of Tractograms 2 (SIFT2)[47]. Connections were then combined into an 82 x 82 undirected and weighted matrices. SIFT2 was chosen in preference to SIFT as it can retain the full connectome and requires significantly less processing time.

Graph metrics were calculated using the brain connectivity toolbox (version 2016-16-01)[48] and have been detailed elsewhere[49]. We measured connection strength of the 6 striatal regions, 12 cortical rich club regions and whole brain network measures of modularity and global efficiency.

The strength of each connection is calculated by the sum of its connection weights. The 12 cortical rich club regions selected were those with the highest connection strength in the network. These were the superior frontal, precentral, superior parietal, thalamus, inferior parietal and rostral middle frontal regions from both hemispheres. The rich club regions were the same for gene carriers and controls and consistent with previous literature[40,50].

Whole brain connectivity was assessed by using measures of modularity and global efficiency. Average path length represents the average of shortest paths between brain regions in a network. Global efficiency is the inverse of the average shortest path length and a decrease represents loss of network integration. Modularity refers to the community structure within brain networks. Modules are clusters of nodes with dense

interconnectivity within the cluster but sparse connections between nodes in different clusters. As modularity increases, the network is more segregated with fewer connections between different modules.

Biofluids

Of the 109 participants with CSF samples, 5 preHD had CSF collected previously under the same collection protocol as part of the HDClarity study (http://hdclarity.net) between -222 and +425 days of their YAS visit. The mean duration of deviation was +61 days. Blood collection was performed between 0930-1030 without fasting, with samples placed on wet ice and processed within 30 minutes of collection. Plasma was isolated by centrifugation and frozen. CSF sample collection and processing were standardised as previously described[51,52]. Lumbar punctures were carried out between 0830-1030 after overnight fasting. Samples were placed on wet ice and processed within 30 minutes of collection by centrifugation and freezing using standard kits containing polypropylene plasticware supplied by the HDClarity study. All samples were stored at -80°C and analysed blinded to disease status and clinical data. Biofluid assays are detailed in Supplementary Table 2. Plasma Ubiquitin Carboxy-terminal Hydrolase L1 (UCH-L1) results were not included in the analysis due to previously reported high variability in assay performance[53,54]. Haemoglobin concentration was measured using a commercial ELISA to determine CSF contamination by blood. Haemoglobin, mutant and total huntingtin (tHTT) levels were measured in triplicate with the other analytes all measured in duplicate. CSF mutant huntingtin (mHTT) quantification was undetectable in healthy controls and 3 gene carriers with low disease burden scores. Otherwise all analytes were above the limit of detection in the study.

NfL trajectories (Figure 4) were produced by combining data from HD-YAS and HD-CSF[55]. All HD-YAS NfL data was included in the modelling (58 preHD and 51 controls for CSF and 63 preHD and 67 controls for plasma). HD-CSF had CSF and plasma NfL data from 40 manifest subjects, 20 preHD and 20 controls. These subjects were older than the HD-YAS cohort with average ages and standard deviations of 50.7 +/- 11.0, 42.4 +/- 11.1 and 56.0 +/- 9.4 respectively. Therefore combining the two datasets enabled modelling NfL trajectories from 20-70 years for commonly occurring CAG repeat lengths of 40-45. The mean age of onset for each given CAG was annotated using estimates provided from previously published data of 2913 Huntington's disease individuals using the Langbehn equation[3].

Statistics

The primary analysis model had the generic form:

$$y = b_0 + I_{pHD} * (b_1 + b_2 * CAG + b_3 * age + b_4 * CAG * age) + b_5 * age + b_6 * gender + b_7 * age * gender + b_8 * IQ + e$$

Where y is an outcome variable, I_{pHD} = 1 if a participant is from the preHD group and 0 otherwise, the b_i's are linear regression coefficients, and e is the residual random error term, assumed to be independently, identically normally distributed among the participants with 0 mean. Two-tailed tests were performed.

One of the key outcomes from the inter-extra dimensional shift task, the number of levels passed, is not an appropriate outcome for a conventional linear model. For this outcome, we instead used an ordinal logistic regression model with the same covariates noted above.

We repeated all cognitive analyses with the removal of the National Adult Reading Test (NART) as a covariate and repeated all imaging analyses with the removal of intracranial volume as a covariate.

All CSF and serum measures were analysed after log transformation, given the skewed empirical distributions of the raw data. All analytes, with the exception of total huntingtin, Glial Fibrillary Acidic Protein (GFAP) and UCH-L1, were considered primary for the analysis and Benjamini-Hochberg false discovery rate (FDR) estimates are reported together with p values, the hypothesis tests being arguably positive-regression-dependent (i.e., the probability of finding any false positive result does not decrease the probability of finding any other false positive result.) To test possible contamination by blood, CSF haemoglobin concentration was measured and for any CSF analyte with a significant association with haemoglobin, we repeated the model with haemoglobin levels included as an additional candidate covariate.

More than 10% of the combined case and control CSF data was missing, primarily due to participants declining to undergo the optional lumbar puncture. Subsequently, we used multiple imputations to estimate CSF-model parameter estimates and associated hypothesis test results[56]. We generated 20 imputations via random forest predictions (10 trees) using the joint empirical distribution of all log-transformed CSF and plasma measures, along with age, sex, and control versus preHD participant status. We generated 40 imputations of the data and final model estimates and p values were derived using Rubin's rules.

Within the preHD group, we first assessed for unadjusted correlations between mHTT and NfL. We then determined how well the clinical, neuroimaging and biofluid variables are predicted by CSF levels of mutant huntingtin and by CSF and plasma NfL. Analyses was performed one biomarker at a time, with FDR correction, with the general form;

$$\gamma = b_0 + b_1 * biomarker + b_2 * age + b_3 * gender + b_4 * age * gender + b_5 + IQ + e$$

We used nonparametric bootstrapping to test for a significant preHD versus control difference in relative caudate and putamen volume loss. In each bootstrap replication, we separately fit regression models for caudate and putamen volumes. After controlling for age and sex, we calculated the adjusted preHD versus control ratios of both caudate and putamen volumes. The null hypothesis is that these caudate and putamen ratios are equal. Our bootstrapped statistic was the ratio of these two ratios, which would equal 1 under the null. We calculated two-sided p-values by inverting bias corrected accelerated-corrected interval estimates from a set of 5,000 bootstrap replications.

A sensitivity analysis was performed to examine the effect of two outliers in the putaminal volume analysis, refitting the relevant model with the outliers removed.

We constructed CAG-specific models of age and NfL concentrations using backwards variable elimination from a saturated two-degree polynomial models of main effects and interactions of age and CAG-length.

Statistical software

All analyses were performed in R (versions 3.5.1 and 3.5.3). SAP models were fit via the lm() function, and we calculated false discovery rates via the p.adjust() function with option 'BH'. We used the boot library for model-comparison boot-strapping and the mouse library for multiple imputation generation and summarization. We used a combination of the ggROC and pROC packages to generate receiver operating characteristic curves and to calculate associated summary statistics.

Results

Research genotyping

Research genotyping was performed to confirm CAG expansion and standardise CAG sizing for subsequent analysis. Only five gene carriers had a subsequent change in CAG size with four ±1 and one +3 CAG repeats.

Cognition

Standardised means with confidence intervals (CIs) for all cognitive variables are shown in Supplementary Table 3. The IED task showed an uncorrected group effect (p=0.04) in ED errors, although this did not survive FDR correction for multiple comparisons (FDR=0.28). The RVP task showed some evidence of impaired performance in the preHD group (p=0.05) but this did not survive FDR correction (FDR=0.28). Emotion processing in the intensity decreasing task was slightly impaired in the preHD group although this did not survive correction with FDR (p=0.05; FDR=0.28). Semantic verbal fluency also showed some reduced

performance in the preHD group (p=0.09; FDR = 0.35). No further variables showed any notable evidence of group effects.

The only cognitive task with some association with age-by-CAG was the breakpoint in the progressive ratio but this was not significant at the FDR level (p=0.005; FDR=0.13).

Results were statistically unchanged when the NART was removed as a covariate.

Neuropsychiatry

Standardised means with CIs for all neuropsychiatric variables are shown in Supplementary Table 4. None of the variables showed notable evidence of group effects.

Imaging

Mean differences and CIs between preHD and controls for all volumetric imaging measures are shown in Supplementary Table 5. There were no significant associations between volumetric measures and age and CAG (FDR>0.48).

Axial diffusivity in the genu of the corpus callosum was lower in the preHD group than controls (p=0.007), although this did not survive correction for FDR (FDR=0.30) (Supplementary Table 6). The Isotropic volume fraction in this region also showed uncorrected group differences (p=0.04; FDR=0.54). In addition, the orientation dispersion in the posterior limb of the internal capsule was reduced in the preHD group (p=0.04) but this did not survive FDR correction (FDR=0.54). There were no associations with age-by-CAG for any of the diffusion or NODDI measures.

There was increased R1 signal in the putamen in preHD compared with controls (p=0.005), although this did not survive FDR correction (FDR=0.17) (Supplementary Table 7). There was also an uncorrected group difference in R1 signal in the external capsule which did not survive FDR correction (p=0.05; FDR=0.29). R2 signal was elevated in the posterior internal capsule (p=0.02), the external capsule (p=0.02) and the putamen (0.03) in preHD; none of these results survived correction for FDR (FDR> 0.20). There were no significant associations with age-by-CAG for any of the MPM measures.

There were no significant group differences in structural connectivity measures (Supplementary Table 8). Structural connectivity measures did not show any association with age-by-CAG.

Results were similar when total intracranial volume was removed as a covariate.

Biofluids

Mean differences between preHD and controls for all biofluid measures are shown in Supplementary Table 9. CSF haemoglobin was associated with mHTT and YKL-40, and was controlled for in the subsequent analyses. CSF haemoglobin was not associated with any other analyte. Only CSF NfL was significantly predicted by age-by-CAG, with higher age and CAG associated with higher NfL levels (FDR=<0.0001).

One control outlier for CSF NfL was noted. They had a normal T1 brain scan, normal CSF white cell and red cell counts and did not outlie in other biofluid or cognitive parameters and so was not excluded from the analysis.

mHTT and NfL predictions of other Huntington's disease measures

CSF NfL was highly correlated with plasma NfL and CSF mHTT (r=0.68 and 0.57; p=<0.0001) whilst plasma NfL also correlated with mHTT to a lesser extent (r=0.31, p=0.02). There were significant associations between CSF mHTT and CSF YKL-40, CSF Tau, and CSF UCH-L1 (r =0.54, 0.50 and 0.50 respectively, FDR=0.003). CSF NfL correlated with CSF YKL-40 (r=0.37; FDR=0.04).

NfL and mHTT did not significantly correlate with any imaging, cognitive, or neuropsychiatric measure.

Discussion

Cognition

The ED set shifting stage has previously been shown to be sensitive to Huntington's disease, both in early[57] and in the preHD stage up to 10 years prior to onset[58]. A recent study in obsessive-compulsive disorder demonstrated that EDS errors were associated with a specific frontal-striatal pathway between the ventrolateral prefrontal cortex and the caudate[59]; this pathway is known to be vulnerable to Huntington's disease pathology. The group differences found in the present study are consistent with these previous findings, despite the failure to achieve a low false discovery rate. This may be due to very subtle impairment in a few individuals closer to disease onset. Similarly there was some evidence of impaired sustained attention in the preHD group as measured by A' on the RVP.

Lawrence et al[58] previously reported significant deficits in semantic verbal fluency in preHD and again the subthreshold group difference in this task may be suggestive of a small subset of individuals showing very mild impairment.

Impaired performance on the OTS has been shown in early Huntington's disease patients[60] and a study by Pavese et al[61] demonstrated an association between accuracy on this test and striatal D2 receptor binding. However, another study in preHD found no significant impairments in the OTS[58] which is consistent with the results that we report here, suggesting that impairment in this domain occurs later in the disease process.

The results on the intensity morphing task indicated bias towards negative emotional faces.

The PAL test is a good indicator of mild cognitive impairment[62] and performance is strongly associated with the hippocampus[63]. There is evidence of impaired cognitive performance on the PAL in early but not preHD[64]. We found no impairment on this task and no association with age-by-CAG.

Although individuals with early Huntington's disease have been shown to have impaired performance on the SWM[65], a preHD cohort did not appear show any deficits[58], which is consistent with the results from our cohort.

Tabrizi et al[66] demonstrated the SDMT to be impaired in preHD and Paulsen and Long[67] showed a progressive decline in performance as years to disease onset decreased. However, this task did not prove sensitive in our very early preHD cohort.

Heath et al[6] showed decreased progressive ratio breakpoints in both humans and rodents with Huntington's disease. Moreover, in humans the breakpoint scores were significantly correlated with self-report measures of apathy.

Imaging

Sensitivity analysis revealed that the results were largely unchanged by removal of the two outliers with small putaminal volumes, suggesting that the finding of reduced volumes of the putamen in gene carriers is robust.

Apart from the striatal volume reduction discussed in the main text, there is no robust evidence that carrying the gene is having a substantial impact on brain volume, microstructure or structural connectivity at this stage of the disease. The lack of association between these measures and age-by-CAG suggests that there is not a particularly strong disease effect in this cohort. Nevertheless, it is possible that there are some subtle changes occurring which are suggestive of the start of the neurodegenerative process. The subthreshold changes in the putamen in R1 and R2 signal in the absence of changes in MT may suggest that iron is starting to accumulate, which would be consistent with evidence of elevated iron in established disease[68].

Biofluids

We report elevations in YKL-40, a marker of astrocytic activation, in the preHD group. The absence of corresponding elevations in GFAP, another marker of astrocytic activation, may reflect the limited nature of astrocyte activation at this stage and/or its relative insensitivity as a biomarker, consistent with findings in AD cohorts[69]. This is the first study of UCH-L1 in Huntington's disease. UCH-L1 is an abundant cytoplasmic protein in the central nervous system, making up to 1-5% of total soluble protein and is elevated in traumatic brain injury and neurodegenerative disease[53,54]. The non-significant findings here support the notion that there is no widespread neuronal damage at this stage of Huntington's disease consistent with imaging findings.

The pro-inflammatory cytokines IL-6 and IL-8, previously found to be elevated in the serum in preHD, were not elevated in the CSF in our cohort. This is in keeping with prior findings by Rodrigues et al[70]. Recent novel findings suggest the possibility of detecting further evidence of atypical immune activity in CSF before onset in Huntington's disease, if validated, may be worthy of study in this population[71].

Consistent with previous reports[55], NfL was closely associated with CSF mHTT suggesting that both are released together from damaged axons. Despite there being no significant group changes in Tau and UCH-L1, both were positively associated with mHTT (FDR=0.003 respectively). These associations suggest that there may be a shared pathway in which mHTT, Tau and UCH-L1 are released from neurons in preHD. The absence of other significant associations between NfL, mHTT and other imaging, cognitive or neuropsychiatric measure strengthens our null results in the latter domains.

Supplementary Table 1: Number of assessments by modality

Full datasets were available for all modalities with the following exceptions

Assessment	Gene Carriers (N=64)	Controls (N=67)
**T1 Volumetric**	62	61
**DTI**	60	60
**NODDI**	60	60
**MPMs (all acquisitions)**	57	59
**MPM R2* only**	57	56
**Structural Connectivity**	54	53
**Plasma**	63	67
**CSF**	58	51

Supplementary Table 2: Biofluid assay details

Analyte	Source	Assay name	Platform	Manufacturer	Performed by	Median CV	N CV >20%
**mHTT**	CSF	2B7-MW1 immunoassay	SMC™ Erenna®	Singulex	Evotek	9.9	6
**tHTT**	CSF	2B7-DF7 immunoassay	SMC™ Erenna®	Singluex	IRBM	9.2	10
**NfL**	CSF	Neurology 4-Plex A	SIMOA HD-1 Analyzer™	Qunaterix	UCL	2.5	0
**NfL**	Plasma	Neurology 4-Plex A	SIMOA HD-1 Analyzer™	Qunaterix	UCL	4.3	2
**Tau**	CSF	Neurology 4-Plex A	SIMOA HD-1 Analyzer™	Qunaterix	UCL	2.6	0
**Tau**	Plasma	Neurology 4-Plex A	SIMOA HD-1 Analyzer™	Qunaterix	UCL	4.1	2
**GFAP**	CSF	Neurology 4-Plex A	SIMOA HD-1 Analyzer™	Qunaterix	UCL	2.0	0
**UCH-L1**	CSF	Neurology 4-Plex A	SIMOA HD-1 Analyzer™	Qunaterix	UCL	4.8	2
**IL-6**	CSF	IL-6	SIMOA HD-1 Analyzer™	Qunaterix	UCL	2.4	0
**IL-8**	CSF	IL-8	SIMOA HD-1 Analyzer™	Qunaterix	UCL	4.7	5
**Neurogranin**	CSF	Neurogranin	ELISA	Euroimmun	UCL	3.5	2
**YKL-40**	CSF	Human YKL-40 Assay	U-PLEX®	MSD	UCL	1.8	2

Coefficient of Variability; SMC = Single Molecule Counting, MSD = Mesoscale Discovery; SIMOA = Single Molecule Array

Supplementary Table 3: Cognitive Results

Outcome Measure	Controls Mean	HD Mean	Mean Difference (CL)	Effect size (CL)	t	p	FDR
**IED Pre ED Errors**	5·87	5·64	0·22 (-0·53, 0·98)	0·10 (-0·25, 0·46)	0·59	0·35	0·74
**IED ED Errors**	5·06	7·09	-2·03 (-4·51, 0·44)	-0·29 (-0·64, 0·06)	-1·63	0·13	0·47
**IED ED Reversal Errors**	2·71	5·40	-2·69 (-5·23, -0·15)	-0·37 (-0·73, -0·02)	-2·09	0·01	0·22
**IED Stages completed**	8·92	8·73	0·19 (0·01, 0·38)	0·37 (0·01, 0·72)	2·05	0·1	0·44
**Intensity Morphing Decreasing**	11·7	11·2	0·49 (0·00, 0·98)	0·35 (0·00, 0·71)	1·98	0·05	0·28
**Intensity Morphing Increasing**	10·1	10·3	-0·29 (-0·91, 0·34)	-0·16 (-0·51, 0·19)	-0·91	0·37	0·74
**Moral Emotions Guilt Score**	6·06	6·12	-0·05 (-0·25, 0·14)	-0·10 (-0·45, 0·26)	-0·55	0·58	0·80
**OTS Mean Latency**	11·7	11·8	-0·02 (-0·18, 0·13)	-0·05 (-0·40, 0·30)	-0·28	0·78	0·86
**OTS Problems Solved**	11·5	11·6	-0·11 (-0·80, 0·56)	-0·05 (-0·41, 0·30)	-0·32	0·75	0·86
**PAL Total Errors**	19·3	22·3	-3·05 (-10·4, 4·27)	-0·15 (-0·50, 0·21)	-0·83	0·42	0·80
**Reinforcement Learning Rate**	0·81	0·81	-0·01 (-0·05, 0·04)	-0·04 (-0·39, 0·31)	-0·23	0·82	0·86
**Progressive Ratio Breakpoint**	391	388	3·0 (-34·5, 40·4)	0·03 (-0·32, 0·38)	0·16	0·24	0·58
**RVP A'**	0·93	0·92	0·02 (0·00, 0·03)	0·36 (0·00, 0·71)	2·00	0·05	0·38
**RVP Mean Latency**	445	450	-5·66 (-32·4, 21·0)	-0·07 (-0·43, 0·28)	-0·42	0·68	0·83
**SDMT**	60·7	59·7	1·00 (-2·18, 4·17)	0·11 (-0·24, 0·46)	0·62	0·53	0·80
**SST Proportion of Successful Stops**	0·44	0·43	0·01 (-0·02, 0·04)	0·09 (-0·26, 0·44)	0·50	0·62	0·80

**SST RT**	191	201	-9.57 (-25·1, 5·91)	-0·22(-0·58, 0·14)	-1·22	0·23	0·58
**SST Mean RT on Correct Go Trials**	522	512	9·70 (-23·7, 43·1)	0·10 (-0·25, 0·45)	0·58	0·56	0·80
**Stroop Colour Naming**	87·5	84·0	3·48 (-1·37, 8·34)	0·25 (-0·10, 0·61)	1·42	0·16	0·51
**Stroop Word Reading**	106	104	2·25 (-3·45, 7·96)	0·14 (-0·21, 0·49)	0·78	0·44	0·80
**SWM Between Errors**	69·3	68·2	1·10 (-11·9, 14·1)	0·03 (-0·32, 0·38)	0·17	0·87	0·87
**Verbal Fluency**	25·0	23·3	1·66 (-0·28, 3·60)	0·30 (-0·05, 0·65)	1·69	0·09	0·44

CL = confidence levels; t = t-test; Effect size is the standardised mean difference between premanifest and control groups; FDR = false discovery rate; adj = adusted; IED = Intra-extra Dimensional Set Shifting; OTS = One-touch Stockings of Cambridge; PAL = paired associates learning; RVP = rapid visual processing; A'=measure of target sensitivity; SDMT = symbol digit modality task; SST = stop signal task; RT = reaction time; SWM = spatial working memory

Supplementary Table 4: Neuropsychiatric Results

Outcome Measure	Controls Mean	HD Mean	Mean Difference (CL)	Effect size (CL)	t	p	FDR
**PSQI**	5·02	4·38	0·64 (-0·26, 1·55)	0·25 (-0·10, 0·60)	1·41	0·16	0·36
**BIS**	61·7	60·8	0·95 (-2·66, 4·56)	0·09 (-0·26, 0·44)	0·52	0·60	0·76
**STAI Trait**	40·2	38·8	1·35 (-1·86, 4·56)	0·15 (-0·20, 0·50)	0·83	0·41	0·61
**STAI State**	35·1	33·0	2·01 (-0·95, 4·96)	0·24 (-0·11, 0·59)	1·34	0·18	0·36
**OCI**	10·3	7·79	2·55 (-0·32, 5·41)	0·31 (-0·04, 0·66)	1·76	0·08	0·33
**SDS**	33·2	33·4	-0·15 (-2·81, 2·50)	-0·02 (-0·37, 0·33)	-0·11	0·91	0·91
**AMI**	1·25	1·27	-0·02 (-0·17, 0·13)	-0·04 (-0·39, 0·31)	-0·23	0·82	0·91
**FrsBe Apathy**	27·6	26·4	1·11 (-3·75, 5·98)	0·08 (-0·27, 0·43)	0·45	0·65	0·76
**FrsBe Disinhibition**	29·8	25·3	4·45 (0·15, 8·75)	0·36 (0·01, 0·71)	2·05	0·04	0·31
**FrsBe Executive Functioning**	32·8	29·8	2·92 (-2·49, 8·32)	0·19 (-0·16, 0·54)	1·07	0·29	0·52
**SF36 Physical Functioning**	95·4	98·1	-2·69 (-6·00, 0·62)	-0·28 (-0·64, 0·07)	-1·61	0·11	0·33
**SF36 Physical Health Limitations**	93·5	97·5	-4·06 (-9·31, 1·18)	-0·27 (-0·62, 0·08)	-1·53	0·13	0·33
**SF36 Emotional Limitations**	83·1	92·5	-9·43 (-18·94, 0·09)	-0·35 (-0·70, 0·00)	-1·96	0·05	0·31
**SF36 Energy/Fatigue**	59·6	61·0	-1·29 (-7·30, 4·72)	-0·08 (-0·43, 0·28)	-0·42	0·67	0·76
**SF36 Emotional Wellbeing**	75·3	77·6	-2·28 (-7·56, 3·01)	-0·15 (-0·50, 0·20)	-0·85	0·40	0·61
**SF36 Social Functioning**	87·9	89·5	-1·68 (-7·39, 4·04)	-0·10 (-0·45, 0·25)	-0·58	0·56	0·76
**SF36 Pain**	88·5	93·1	-4·62 (-8·83, -0·40)	-0·38 (-0·73, -0·03)	-2·17	0·03	0·31

**SF36 General Health**	73.0	67.9	5.07 (-0.86, 11.00)	0.30 (-0.05, 0.65)	1.69	0.09	0.33

CL = confidence levels; t = t-test; Effect size is the standardised mean difference between premanifest and control groups; FDR = false discovery rate; PSQI = Pittsburgh Sleep Quality Index; SF36 = 36 item Short Form. FrsBE = Frontal Systems Behavioural Scale; AMI = Apathy Motivation Index; SDS = Zung Self-Rating Depression Scale; OCI = Obsessive-Compulsive Index; STAI = Speilberger State/Trait Anxiety Index; BIS = Barratt Impulsivity Scale.

Supplementary Table 5: Volumetric Results (corrected for intracranial volume)

Outcome Measure	Controls Mean	HD Mean	Mean Difference (CL)	Effect size (CL)	t	p	FDR
**Putamen**	0·66	0·62	0·04 (0·01, 0·06)	0·53 (0·16, 0·89)	2·87	0·005	0·03
**Caudate**	0·50	0·48	0·02 (0·00, 0·04)	0·37 (0·00, 0·74)	2·00	0·05	0·20
**Grey matter**	43·7	43·5	0·16 (-0·44, 0·76)	0·10 (-0·27, 0·46)	0·53	0·60	0·92
**White matter**	34·5	34·6	-0·03 (-0·57, 0·51)	-0·02 (-0·39, 0·35)	-0·11	0·91	0·92
**Whole brain**	80·3	80·2	0·12 (-0·68, 0·92)	0·06 (-0·31, 0·42)	0·30	0·76	0·92
**Ventricles**	0·86	0·88	-0·02 (-0·17, 0·13)	-0·05 (-0·41, 0·32)	-0·24	0·81	0·92

CL = confidence levels; t = t-test; Effect size is the standardised mean difference between premanifest and control groups; FDR = false discovery rate; All measures are expressed as a percentage of intracranial volume

Supplementary Table 6: Diffusion Results

Outcome Measure	Controls Mean	HD Mean	Mean Difference (CL)	Effect size (CL)	t	p	FDR q
**AD CC Genu**	1·27	1·26	0·01 (0·00, 0·02)	0·53 (0·16, 0·91)	2·81	0·006	0·27
**AD CC Spl**	1·33	1·32	0·01 (-0·00, 0·02)	0·28 (-0·10, 0·66)	1·47	0·15	0·75
**AD CC mid**	1·27	1·27	0·00 (-0·01, 0·01)	0·17 (-0·20, 0·55)	0·90	0·37	0·86
**AD IC ant**	1·00	1·00	-0·00 (-0·01, 0·01)	-0·26 (-0·63, 0·11)	-1·37	0·17	0·75
**AD IC post**	1·05	1·05	-0·00 (-0·01, 0·01)	-0·15 (-0·52, 0·23)	-0·74	0·46	0·87
**AD EC**	0·95	0·95	0·00 (-0·01, 0·00)	-0·21 (-0·59, 0·17)	-1·10	0·27	0·75
**FA CC Genu**	0·69	0·69	0·00 (-0·01, 0·01)	-0·01 (-0·30, 0·36)	-0·10	0·95	0·98
**FA CC Spl**	0·75	0·75	-0·00 (-0·01, 0·00)	-0·03 (-0·40, 0·35)	-0·14	0·89	0·98
**FA CC mid**	0·67	0·67	-0·00 (-0·01, 0·01)	-0·07 (-0·45, 0·30)	-0·38	0·70	0·92
**FA IC ant**	0·58	0·58	-0·00 (-0·01, 0·00)	-0·19 (-0·56, 0·19)	-1·00	0·32	0·84
**FA IC post**	0·68	0·68	-0·00 (-0·01, 0·00)	-0·23 (-0·60, 0·15)	-1·20	0·23	0·75
**FA EC**	0·45	0·45	-0·00 (-0·01, 0·00)	-0·25 (-0·62, 0·13)	-1·31	0·19	0·75
**MD CC Genu**	0·64	0·63	0·01 (0·00, 0·01)	0·33 (-0·04, 0·71)	1·75	0·09	0·75
**MD CC Spl**	0·63	0·63	0·01 (-0·00, 0·01)	0·24 (-0·14, 0·61)	1·25	0·21	0·75
**MD CC Mid**	0·65	0·65	0·00 (-0·01, 0·01)	0·13 (-0·25, 0·50)	0·67	0·51	0·87
**MD IC ant**	0·57	0·57	-0·00 (-0·00, 0·00)	-0·09 (-0·46, 0·29)	-0·47	0·64	0·92

**MD IC post**	0.54	0.54	0.00 (-0.00, 0.00)	0.07 (-0.30, 0.45)	0.39	0.70	0.92
**RD CC Genu**	0.32	0.32	0.00 (-0.01, 0.01)	0.12 (-0.25, 0.50)	0.64	0.53	0.87
**RD CC Spl**	0.28	0.28	0·00 (-0·00, 0·01)	0·15 (-0·23, 0·53)	0·79	0.43	0.86
**RD CC Mid**	0.34	0.34	0·00 (-0·01, 0·01)	0·08 (-0·30, 0·46)	0.43	0.67	0.92
**RD IC ant**	0.35	0.35	0·00 (-0·00, 0·01)	0·09 (-0·28, 0·47)	0.48	0.63	0.92
**RD IC post**	0.29	0.29	0·00 (-0·00, 0·01)	0·23 (-0·14, 0·61)	1.23	0.22	0.75
**RD EC**	0.45	0.45	0·00 (-0·00, 0·01)	0·12 (-0·25, 0·50)	0.65	0.54	0.87
**ND CC Genu**	0.62	0.62	0.00 (-0.01, 0.01)	0.02 (-0.36, 0.40)	0.11	0.91	0.98
**NDI CC Spl**	0.67	0.67	-0.00 (-0.01, 0.01)	-0.04 (-0.42, 0.33)	-0.23	0.82	0.98
**NDI CC Mid**	0·64	0·63	0·00 (-0·01, 0·01)	0.16 (-0.22, 0.53)	0.82	0.41	0.86
**NDI IC Ant**	0.63	0.63	-0.00 (-0.01, 0.01)	-0.01 (-0.39, 0.36)	-0.08	0.94	0.98
**NDI IC Post**	0.69	0.70	-0·00 (-0·01, 0·00)	-0·16 (-0·53, 0·22)	-0·82	0·41	0·86
**NDI EC**	0·52	0·52	0·00 (-0·01, 0·00)	-0·06 (-0·44, 0·31)	-0·33	0·74	0·92
**FWF CC Genu**	0·12	0·11	0·01 (0·00, 0·01)	0·40 (0·02, 0·77)	2·10	0·04	0·54
**FWF CC Spl**	0·14	0·13	0·00 (-0·00, 0·01)	0·25 (-0·12, 0·63)	1·34	0·18	0·75
**FWF CC Mid**	0·15	0·15	0·01 (-0·00, 0·01)	0·21 (-0·16, 0·59)	1·12	0·27	0·75

**FWF IC ant**	0·15	0·15	0·01 (-0·00, 0·01)	0·21 (-0·16, 0·59)	1·12	0·27	0·75
**FWF IC post**	0·08	0·08	-0·00 (-0·00, 0·00)	-0·07 (-0·44, 0·31)	-0·35	0·73	0·92
**FWF EC**	0.03	0.03	-0.00 (-0.00, 0.00)	-0.24 (-0.61, 0.14)	-1.24	0.22	0.75
**ODI CC Genu**	0.08	0.08	-0.00 (-0.00, 0.00)	-0.07 (-0.45, 0.30)	-0.39	0.70	0.92
**ODI CC Spl**	0.07	0.07	-0.00 (-0.00, 0.00)	0·15 (-0·23, 0·52)	0·78	0·43	0·86
**ODI CC Mid**	0·09	0·09	0·00 (-0·00, 0·00)	0·02 (-0·35, 0·40)	0·13	0·90	0·98
**ODI IC ant**	0·12	0·11	0·00 (-0·00, 0·00)	0·12 (-0·26, 0·49)	0·62	0·54	0·87
**ODI IC post**	0·11	0·11	0·00 (-0·00, 0·00)	0·40 (0·02, 0·78)	2·09	0·04	0·54
**ODI EC**	0.21	0.20	0.00 (-0.00, 0.00)	0.30 (-0.07, 0.68)	1.60	0.11	0.75

CL = confidence levels; t = t-test; Effect size is the standardised mean difference between premanifest and control groups; FDR = false discovery rate; AD = axial diffusivity; MD = mean diffusivity; RD = radial diffusivity; FA = fractional anisotropy; NDI = neurite density index; MD = mean diffusivity; ODI = orientation dispersion index; FWF = free water fraction CC = corpus callosum; IC = internal capsule; EC = external capsule; ant = anterior; post = posterior; mid = mid-body; Spl = splenium

Supplementary Table 7: MPM Results

Outcome Measure	Controls Mean	HD Mean	Mean Difference (CL)	Effect size (CL)	t	p	FDR
**R2* Putamen**	21·5	22·4	-0·86 (-1·65, -0·07)	-0·42 (-0·81, -0·04)	-2·16	0·03	0·20
**R1 Putamen**	0·75	0·77	-0·02 (-0·03, -0·00)	-0·54 (-0·92, -0·15)	-2·75	0·005	0·17
**PD Putamen**	77·9	77·9	-0·01 (-0·70, 0·68)	-0·01 (-0·39, 0·38)	-0·04	0·91	0·98
**MT Putamen**	0·90	0·89	0·01 (-0·00, 0·02)	0·25 (-0·14, 0·63)	1·27	0·21	0·36
**R2* Caudate**	18·7	19·2	-0·49 (-1·06, 0·07)	-0·34 (-0·73, 0·05)	-1·73	0·09	0·36
**R1 Caudate**	0·71	0·72	-0·01 (-0·02, 0·00)	-0·38 (-0·76, 0·01)	-1·93	0·06	0·36
**PD Caudate**	80·0	80·2	-0·22 (-0·68, 0·23)	-0·19 (-0·57, 0·20)	-0·96	0·34	0·78
**MT Caudate**	0·83	0·83	0·01 (-0·00, 0·02)	0·24 (-0·14, 0·63)	1·26	0·21	0·38
**R2* CC Spl**	23·3	23·5	-0·19 (-0·74, 0·37)	-0·13 (-0·52, 0·26)	-0·67	0·51	0·78
**R1 CC Spl**	1·02	1·03	-0·01 (-0·02, 0·01)	-0·13 (-0·51, 0·26)	-0·66	0·51	0·99
**PD CC Spl**	68·6	68·7	-0·03 (-0·29, 0·23)	-0·04 (-0·43, 0·34)	-0·22	0·83	0·98
**MT CC Spl**	1·58	1·59	-0·01 (-0·04, 0·03)	-0·08 (-0·46, 0·31)	-0·40	0·69	0·98
**R2* CC Genu**	22·3	22·5	-0·19 (-0·69, 0·31)	-0·15 (-0·54, 0·24)	-0·76	0·45	0·78
**R1 CC Genu**	1·07	1·08	-0·01 (-0·03, 0·01)	-0·21 (-0·60, 0·17)	-1·11	0·27	0·78
**PD CC Genu**	67.8	68.0	-0.17 (-0.46, 0.12)	-0.22 (-0.61, 0.16)	-1.14	0.26	0.67
**MT CC Genu**	1·67	1·66	0·01 (-0·02, 0·04)	0·13 (-0·25, 0·52)	0·69	0·49	0·67
**R2* CC mid**	20·6	20·8	-0·24 (-0·54, 0·05)	--0·32, (-0·71, 0·07)	-1·62	0·11	0·39

**R1 CC mid**	1·01	1·02	-0·01 (-0·03, 0·00)	-0·31 (-0·70, 0·07)	-1·61	0·11	0·59
**PD CC mid**	68·7	68·8	-0·08 (-0·31, 0·15)	-0·14 (-0·52, 0·25)	-0·71	0·48	0·85
**MT CC mid**	1·55	1·55	-0·00 (-0·03, 0·02)	-0·07 (-0·45, 0·32)	-0·34	0·73	0·98
**R2* IC pos**	20·4	20·8	-0·46 (-0·89, -0·05)	-0·43 (-0·82, -0·04)	-2·20	0·03	0·20
**R1 IC pos**	1·00	1·01	-0·01 (-0·03, 0·01)	-0·21 (-0·60, 0·17)	-1·10	0·28	0·78
**PD IC pos**	67·6	67·7	-0·09 (-0·42, 0·24)	-0·11 (-0·49, 0·28)	-0·56	0·58	0·78
**MT IC pos**	1·52	1·52	0·00 (-0·03, 0·03)	0·01 (-0·38, 0·39)	0·05	0·96	0·84
**R2* IC ant**	22·5	22·5	-0·03 (-0·50, 0·44)	-0·03 (-0·41, 0·36)	-0·13	0·90	0·99
**R1 IC ant**	1·00	1·01	-0·01 (-0·03, 0·00)	-0·29 (-0·67, 0·10)	-1·46	0·15	0·67
**PD IC ant**	69·7	69·8	-0·03 (-0·39, 0·32)	-0·04 (-0·43, 0·34)	-0·22	0·83	0·98
**MT IC ant**	1·49	1·47	0·02 (-0·01, 0·04)	0·23 (-0·15, 0·62)	1·20	0·23	0·36
**R2* EC**	18·5	18·8	-0·32 (-0·61, -0·03)	-0·43 (-0·82, -0·04)	-2·19	0·03	0·20
**R1 EC**	0·89	0·91	-0·02 (-0·03, -0·00)	-0·40 (-0·79, -0·02)	-2·06	0·04	0·29
**PD EC**	71·1	71·0	0·11 (-0·38, 0·60)	0·09 (-0·30, 0·47)	0·44	0·66	0·81
**MT EC**	1·37	1·38	-0·01 (-0·03, 0·02)	-0·09 (-0·47, 0·30)	-0·46	0·65	0·98

CL = confidence levels; t = t-test; Effect size is the standardised mean difference between premanifest and control groups; FDR = false discovery rate; CC = corpus callosum; IC = internal capsule; EC = external capsule; ant = anterior; post = posterior; mid = mid-body; Spl = splenium. R1 = longitudinal relaxation rate; R2* = the effective transverse relaxation rate; MT = magnetization transfer. PD = proton density.

Supplementary Table 8: Structural Connectivity Results

Outcome Measure	Controls Mean	HD Mean	Mean Difference (CL)	Effect Size (CL)	t	p	FDR
**Left Limbic**	0·01	0·01	0·00 (0·00, -0·00)	-0·31 (-0·71, 0·08)	-1·56	0·12	0·77
**Right Limbic**	0·01	0·01	0·00 (0·00, -0·00)	-0·05 (-0·45, 0·34)	-0·25	0·80	0·99
**Left Executive**	0·01	0·01	0·00 (0·00, -0·00)	0·01 (-0·38, 0·40)	0·05	0·96	0·99
**Right Executive**	0·01	0·01	0·00 (-0·00, 0·00)	0·07 (-0·32, 0·47)	0·37	0·71	0·99
**Left Sensorimotor**	0·01	0·01	0·00 (-0·00, 0·00)	0·00 (-0·39, 0·40)	0·01	0·99	0·99
**Right Sensorimotor**	0·01	0·01	0·00 (-0·00, 0·00)	-0·33 (-0·72, 0·07)	-1·64	0·11	0·77
**Left Superior Frontal**	0·05	0·05	0·00 (-0·00, 0·00)	0·01 (-0·38, 0·41)	0·07	0·94	0·99
**Right Superior Frontal**	0·05	0·05	-0·00 (-0·00, 0·00)	-0·19 (-0·58, 0·21)	-0·92	0·36	0·87
**Left Precentral**	0·04	0·04	-0·00 (-0·00, 0·00)	-0·02 (-0·41, 0·37)	-0·10	0·92	0·99
**Right Precentral**	0·04	0·04	-0·00 (-0·00, 0·00)	-0·21 (-0·60, 0·19)	-1·05	0·30	0·87
**Left Superior Parietal**	0·03	0·03	-0·00 (-0·00, 0·00)	-0·17 (-0·57, 0·22)	-0·86	0·39	0·87
**Right Superior Parietal**	0·03	0·03	0·00 (-0·00, 0·00)	0·12 (-0·28, 0·51)	-0·59	0·56	0·99
**Left Thalamus**	0·03	0·03	-0·00 (-0·00, 0·00)	-0·03 (-0·43, 0·36)	-0·18	0·86	0·99
**Right Thalamus**	0·03	0·03	0·00 (-0·00, 0·00)	0·21 (-0·19, 0·60)	1·05	0·29	0·87
**Left Inferior Parietal**	0·02	0·02	0·00 (-0·00, 0·00)	0·19 (-0·20, 0·59)	0·97	0·33	0·87
**Right Inferior Parietal**	0·03	0·03	0·00 (-0·00, 0·00)	0·12 (-0·28, 0·51)	0·60	0·54	0·99

**Left Rostral Middle Frontal**	0·02	0·02	0·00 (0·00, 0·00)	0·42 (0·03, 0·82)	2·11	0·04	0·71
**Right Rostral Middle Frontal**	0.02	0.02	0.00 (-0.00, 0.00)	-0.02 (-0.41, 0.38)	-0.10	0.92	0.99
**Efficiency**	0.00	0.00	-0.00 (-0.00 0.0)	-0.02 (-0.41, 0.38)	-0.10	0.92	0.99
**Modularity**	0.46	0.46	0.00 (-0.00, 0.01)	0.25 (-0.14, 0.65)	1.27	0.20	0.87

Efficiency is a measure of network integration whilst Modularity is a measure of network segregation. All other measures represent connection strength of each respective region. Limbic, executive and sensorimotor are divisions of the striatum. Other measures are cortical hub regions. CL = confidence levels; Effect size is the standardised mean difference between premanifest and control groups.

Supplementary Table 9: Biofluid Results

Outcome Measure	Controls Mean	HD Mean	Mean Difference (CL)	Effect Size (CL)	t	p	FDR
**NfL CSF**	5·79	6·40	-0·61 (-0·82, -0·42)	-1·17 (-1·56, -0·79)	-6·03	<0·0001	<0·0001
**NfL plasma**	2·02	2·28	-0·26 (-0·43, -0·09)	-0·55 (-0·90, -0·20)	-3·09	0·003	0·01
**YKL-40 CSF**	10·97	11·12	-0·15 (-0·26, 0·03)	-0·50 (-0·89, -0·12)	-2·56	0·01	0·03
**IL6 CSF**	1·01	0·98	0·03 (-0·14, 0·20)	0·07 (-0·31, 0·46)	0·37	0·68	0·77
**IL8 CSF**	4·14	4·19	-0·05 (-0·19, 0·08)	-0·15 (-0·54, 0·23)	-0·79	0·41	0·54
**Neurogranin CSF**	5·68	5·68	0·00 (-0·15, 0·15)	0·00 (-0·38, 0·39)	0·02	0·94	0·94
**Tau CSF**	4·16	4·22	-0·06 (-0·18, 0·06)	-0·20 (-0·58, 0·19)	-1·01	0·34	0·54
**Tau plasma**	1·57	1·44	0·13 (-0·09, 0·34)	0·20 (-0·14, 0·56)	1·14	0·26	0·52
**UCH-L1 CSF**	7·14	7·17	-0·03 (-0·11, 0·05)	-0·16 (-0·55, 0·22)	-0·83	0·41	0·48
**GFAP CSF**	9·43	9·37	-0·06 (-0·10, 0·22)	0·14 (-0·25, 0·52)	0·72	0·48	N/A
**GFAP Plasma**	4·38	4·51	-0·13 (-0·33, 0·07)	-0·23 (-0·58, 0·12)	-1·28	0·20	N/A
**tHTT CSF (log fM)**	3·85	3·75	0·09 (-0·06, 0·25)	0·24 (-0·15, 0·62)	1·23	0·22	N/A
**mHTT CSF (log fM)**	0	3·08	N/A	N/A	N/A	N/A	N/A

All values in log pg/ml unless otherwise stated. UCH-L1, GFAP and tHTT were designated exploratory analyses and therefore not FDR corrected. CL = confidence levels; Effect size is the standardised mean difference between premanifest and control groups.

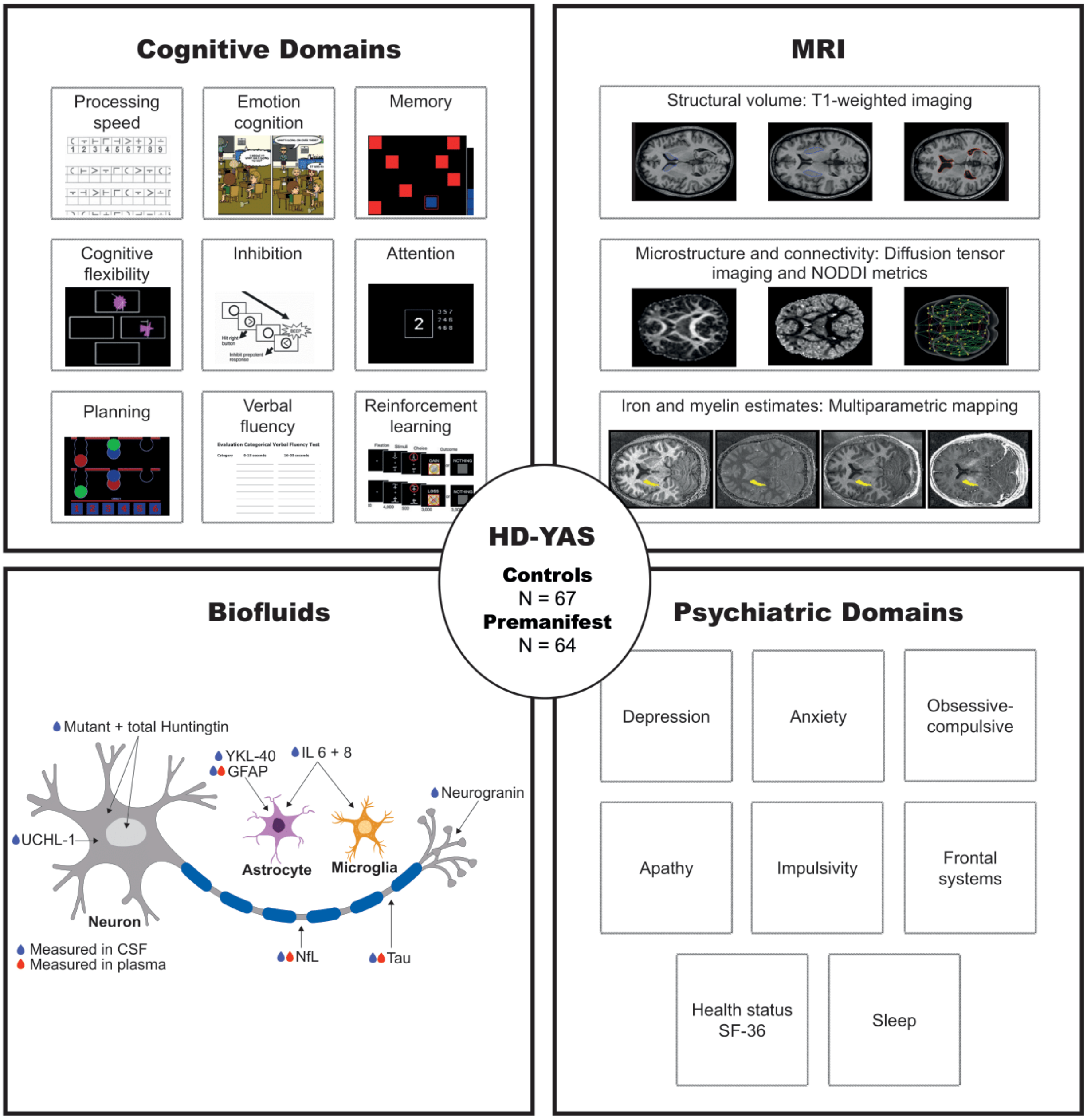

Supplementary Figure 1: Overview of the HD Young Adult study assessments within each domain

Cognitive assessment included tasks from the CANTAB and EMOTICOM batteries to study multiple cognitive domains. Multi-modal MRI was performed to investigate white and grey matter macro and microstructure and connectivity. Psychiatric domains were measured using validated self-report questionnaires. Several biofluid biomarkers were measured in CSF and plasma. Study assessments are described in detail in supplementary material.

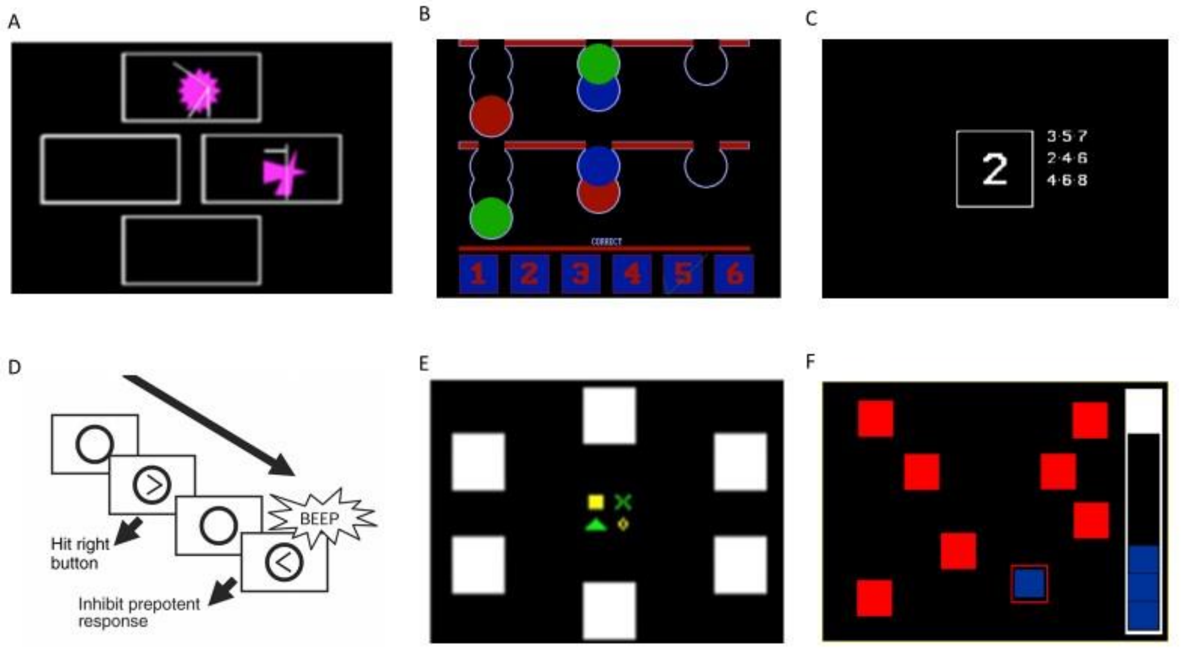

Supplementary Figure 2: Composite image of the CANTAB tests

A) CANTAB Intra-extra dimensional set shifting; B) CANTAB One Touch Stockings of Cambridge; C) CANTAB Rapid Visual Informational Visual Processing; D) CANTAB Stop Signal Test; E) CANTAB Paired Associate Leaning; F) CANTAB Spatial Working Memory.

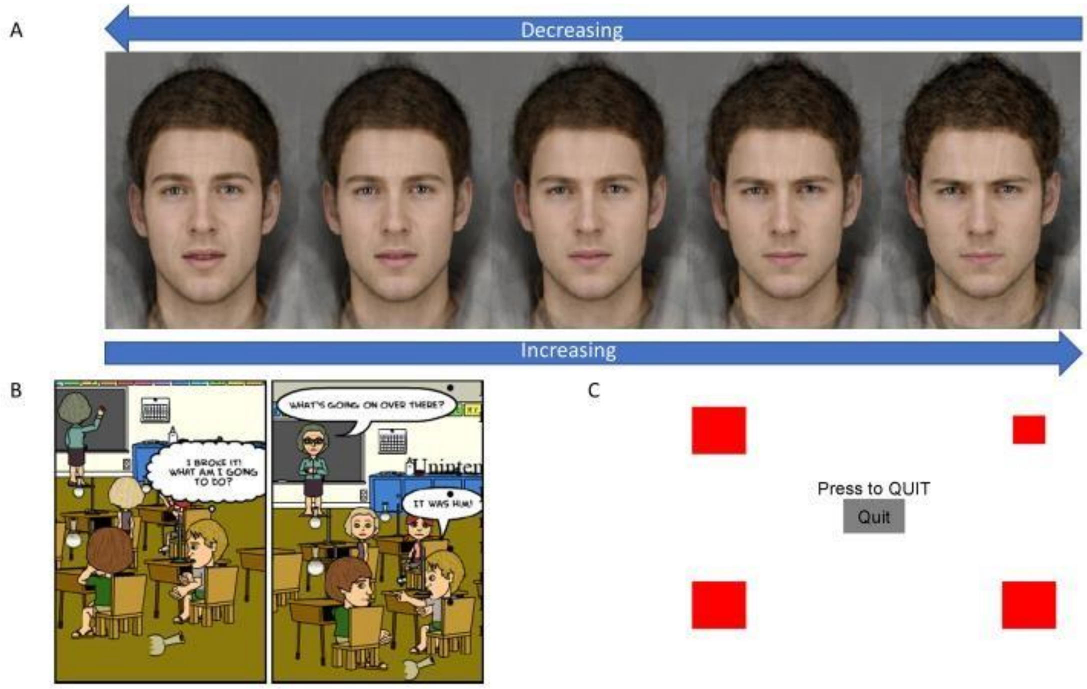

Supplementary Figure 3: Composite image of the EMOTICOM tests

A) EMOTICOM Emotional Intensity Face Morphing; B) EMOTICOM Moral Judgement; C) EMOTICOM Progressive Ratios

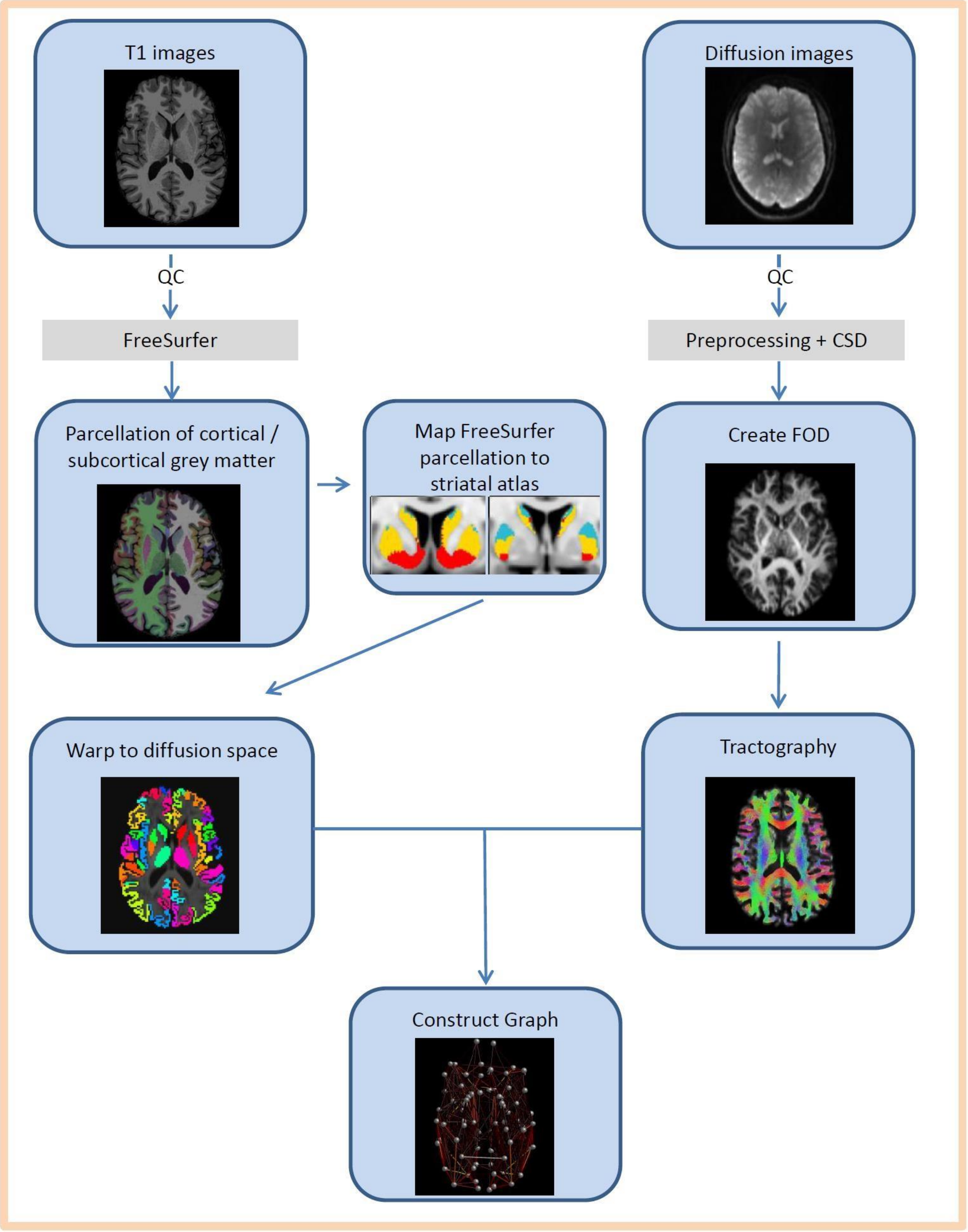

Supplementary Figure 4: Summary of connectivity processing pipeline

QC = quality control. Preprocessing = Eddy current and motion correction, bias correction. CSD = Multi-shell multi-tissue constrained spherical deconvolution. FOD = fibre orientation distributions. Striatal atlas reprinted from FSL https://fsl.fmrib.ox.ac.uk/fsl/fslwiki/Atlases/striatumconn

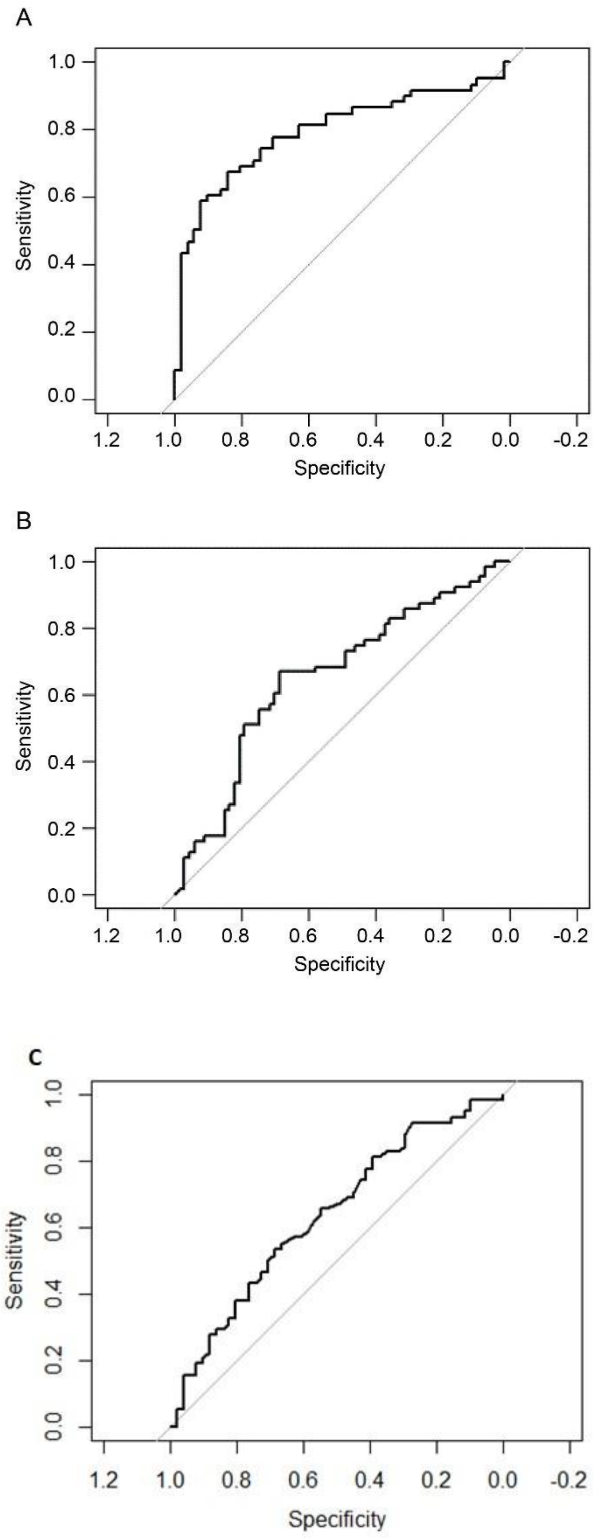

Supplementary Figure 5: ROC curves for NfL and YKL-40

CSF (A) and plasma (B). NfL gave an area under the curve of 0.79 in CSF and 0.65 plasma, implying superior sensitivity/specificity of CSF NfL over plasma NfL in those far from predicted clinical onset in preHD. CSF YKL-40

gave an area under the curve of 0.64, implying inferior sensitivity/specificity as compared to both CSF and plasma NfL.

References

1 Huntington Study Group. Unified Huntington's Disease Rating Scale: reliability and consistency. *Mov Disord* 1996; **11**: 136–42.

2 Penney JB, Vonsattel JP, MacDonald ME, Gusella JF, Myers RH. CAG repeat number governs the development rate of pathology in Huntington's disease. *Ann Neurol* 1997; **41**: 689–92.

3 Langbehn DR, Brinkman RR, Falush D, Paulsen JS, Hayden MR. A new model for prediction of the age of onset and penetrance for Huntington's disease based on CAG length. *Clin Genet* 2004; **65(4):** 267-77.

4 Barker RA, Mason SL, Harrower TP, *et al.* The long-term safety and efficacy of bilateral transplantation of human fetal striatal tissue in patients with mild to moderate Huntington's disease. *J Neurol Neurosurg Psychiatry* 2013; **84(6):** 657-65.

5 Bland AR, Roiser JP, Mehta MA, *et al.* EMOTICOM: A neuropsychological test battery to evaluate emotion, motivation, impulsivity, and social cognition. *Front Behav Neurosci* 2016; **10**: 25.

6 Heath CJ, O'Callaghan C, Mason SL, *et al.* A Touchscreen Motivation Assessment Evaluated in Huntington's Disease Patients and R6/1 Model Mice. *Front Neurol* 2019; **10:** 858.

7 Pessiglione M, Seymour B, Flandin G, Dolan RJ, Frith CD. Dopamine-dependent prediction errors underpin reward-seeking behaviour in humans. *Nature* 2006; **442(7106):** 1042-5.

8 Palminteri S, Justo D, Jauffret C, *et al.* Critical Roles for Anterior Insula and Dorsal Striatum in Punishment-Based Avoidance Learning. *Neuron* 2012; **76(5):** 998-1009.

9 Zung WWK, Richards CB, Short MJ. Self-Rating Depression Scale in an Outpatient Clinic: Further Validation of the SDS. *Arch Gen Psychiatry* 1965; **13(6):** 508-15.

10 Spielberger CD, Gorsuch RL, Lushene RE. STAI manual for the state-trait anxiety inventory. Self-Evaluation Questionnaire. 1983; Consulting Psychologists Press (Palo Alto, CA).

11 Patton JH, Stanford MS, Barratt ES. Factor structure of the barratt impulsiveness scale. *J Clin Psychol* 1995; **51(6):** 631-42.

12 Stout JC, Ready RE, Grace J, Malloy PF, Paulsen JS. Factor analysis of the Frontal Systems Behavior Scale (FrSBe). *Assessment* 2003; **10(1):** 79-85.

13 Foa EB, Huppert JD, Leiberg S, *et al.* The obsessive-compulsive inventory: Development and validation of a short version. *Psychol Assess* 2002; **14(4):** 485-96.

14 Ang YS, Lockwood P, Apps MAJ, Muhammed K, Husain M. Distinct subtypes of apathy revealed by the apathy motivation index. *PLoS One* 2017; **12(1):** e0169938.

15 Sockeel P, Dujardin K, Devos D, Denève C, Destée A, Defebvre L. The Lille apathy rating scale (LARS), a new instrument for detecting and quantifying apathy: Validation in Parkinson's disease. *J Neurol Neurosurg Psychiatry* 2006; **77(5):** 579-85.

16 Buysse DJ, Reynolds CF, Monk TH, Berman SR, Kupfer DJ. The Pittsburgh sleep quality index: A new instrument for psychiatric practice and research. *Psychiatry Res* 1989; **28(2):** 193-213.

17 Ware JE, Sherbourne CD. The MOS 36-item short-form health survey (Sf-36): I. conceptual framework and item selection. *Med Care* 1992; **30(6)**: 473-83.

18 Auerbach EJ, Xu J, Yacoub E, Moeller S, Uğurbil K. Multiband accelerated spin-echo echo planar imaging with reduced peak RF power using time-shifted RF pulses. *Magn Reson Med* 2013; **69(5):** 1261-7.

19 Sled JG, Zijdenbos AP, Evans AC. A comparison of retrospective intensity non-uniformity correction methods for MRI. In: Lecture Notes in Computer Science (including subseries Lecture Notes in Artificial Intelligence and Lecture Notes in Bioinformatics). 1997.

20 Freeborough PA, Fox NC, Kitney RI. Interactive algorithms for the segmentation and quantitation of 3-D MRI brain scans. *Comput Methods Programs Biomed* 1997; **53**: 15–25.

21 Fox NC, Freeborough PA, Rossor MN. Visualisation and quantification of rates of atrophy in Alzheimer's disease. *Lancet* 1996; **348(9020):** 94-7.

22 Whitwell JL, Crum WR, Watt HC, Fox NC. Normalization of cerebral volumes by use of intracranial volume: Implications for longitudinal quantitative mr imaging. *Am J Neuroradiol* 2001; **22**: 1483–9.

23 Ledig C, Heckemann RA, Hammers A, *et al.* Robust whole-brain segmentation: application to traumatic brain injury. *Med Image Anal* 2015; **21**: 40–58.

24 Ashburner J, Friston KJ. Voxel-based morphometry--the methods. *Neuroimage* 2000; **11**: 805–21.

25 Jenkinson M, Bannister P, Brady M, Smith S. Improved optimization for the robust and accurate linear registration and motion correction of brain images. *Neuroimage* 2002; **17**: 825–41.

26 Andersson JLR, Sotiropoulos SN. An integrated approach to correction for off-resonance effects and subject movement in diffusion MR imaging. *Neuroimage* 2016; **141:** 556-72.

27 Graham MS, Drobnjak I, Zhang H. Realistic simulation of artefacts in diffusion MRI for validating post-processing correction techniques. *Neuroimage* 2016: **125**: 1079-94.

28 Zhang H, Schneider T, Wheeler-Kingshott CA, Alexander DC. NODDI: Practical in vivo neurite orientation dispersion and density imaging of the human brain. *Neuroimage* 2012; **61**: 1000–16.

29 Daducci A, Canales-Rodríguez EJ, Zhang H, Dyrby TB, Alexander DC, Thiran JP. Accelerated Microstructure Imaging via Convex Optimization (AMICO) from diffusion MRI data. *Neuroimage* 2015; **105:** 32-44.

30 Zhang J, Gregory S, Scahill RI, *et al.* In vivo characterization of white matter pathology in premanifest huntington's disease. *Ann Neurol* 2018; **84(4):** 497-504.

31 Mori S, Oishi K, Jiang H, *et al.* Stereotaxic white matter atlas based on diffusion tensor imaging in an ICBM template. *Neuroimage* 2008; **40(2):** 570-82.

32 Keihaninejad S, Zhang H, Ryan NS, *et al.* An unbiased longitudinal analysis framework for tracking white matter changes using diffusion tensor imaging with application to Alzheimer's disease. *Neuroimage* 2013; **72:** 153-63.

33 Mahoney CJ, Simpson IJA, Nicholas JM, *et al.* Longitudinal diffusion tensor imaging in rontotemporal dementia. *Ann Neurol* 2015; **77(1):** 33-46.

34 Weiskopf N, Suckling J, Williams G, *et al.* Quantitative multi-parameter mapping of R1, PD*, MT, and R2* at 3T: A multi-center validation. *Front Neurosci* 2013; **7**: 95.

35 Tabelow K, Balteau E, Ashburner J, *et al.* hMRI – A toolbox for quantitative MRI in neuroscience and clinical research. *Neuroimage* 2019; **194:** 191-210.

36 Ourselin S, Roche A, Subsol G, Pennec X, Ayache N. Reconstructing a 3D structure from serial histological sections. Image and Vision Computing . *Image Vis Compting* 2001; **19**: 25–31.

37 Kullmann S, Callaghan MF, Heni M, *et al.* Specific white matter tissue microstructure changes associated with obesity. *Neuroimage* 2016; **125:** 36-44.

38 Desikan RS, Ségonne F, Fischl B, *et al.* An automated labeling system for subdividing the human cerebral cortex on MRI scans into gyral based regions of interest. *Neuroimage* 2006; **31(3):** 968-80.

39 Hibar DP, Stein JL, Renteria ME, *et al.* Common genetic variants influence human subcortical brain structures. *Nature* 2015; **520(7546):** 224-9.

40 Smith RE, Tournier JD, Calamante F, Connelly A. Anatomically-constrained tractography: Improved diffusion MRI streamlines tractography through effective use of anatomical information. *Neuroimage* 2012; **119:** 338-51.

41 McColgan P, Seunarine KK, Razi A, *et al.* Selective vulnerability of Rich Club brain regions is an organizational principle of structural connectivity loss in Huntington's disease. *Brain* 2015; **138**: 3327–44.

42 McColgan P, Seunarine KK, Gregory S, *et al.* Topological length of white matter connections predicts their rate of atrophy in premanifest Huntington's disease. *JCI insight* 2017; **2(8):** 92641.

43 Tziortzi AC, Haber SN, Searle GE, *et al.* Connectivity-based functional analysis of dopamine release in the striatum using diffusion-weighted MRI and positron emission tomography. *Cereb Cortex* 2014; **24(5):** 1165-77.

44 Smith SM, Jenkinson M, Woolrich MW, *et al.* Advances in functional and structural MR image analysis and implementation as FSL. *NeuroImage*. 2004; **23:** Suppl 1: S208-19.

45 Jeurissen B, Tournier JD, Dhollander T, Connelly A, Sijbers J. Multi-tissue constrained spherical deconvolution for improved analysis of multi-shell diffusion MRI data. *Neuroimage* 2014; **103:** 411-426.

46 Tournier J-D, , F. Calamante and a. C. Improved probabilistic streamlines tractography by 2 nd order integration over fibre orientation distributions. *ISMRM* 2010.

47 Smith RE, Tournier JD, Calamante F, Connelly A. SIFT2: Enabling dense quantitative assessment of brain white matter connectivity using streamlines tractography. *Neuroimage* 2015; **119:** 338-51.

48 Rubinov M, Sporns O. Complex network measures of brain connectivity: Uses and interpretations. *Neuroimage* 2010; **52(3):** 1059-69.

49 Bullmore E, Sporns O. Complex brain networks: Graph theoretical analysis of structural and functional systems. Nat. Rev. Neurosci. 2009;**10(4):** 312.

50 Baggio HC, Segura B, Junque C. Resting-State Functional Brain Networks in Parkinson's Disease. CNS Neurosci. Ther. 2015; **21(10):** 793-801.

51 Wild EJ, Boggio R, Langbehn D, *et al.* Quantification of mutant huntingtin protein in cerebrospinal fluid from Huntington's disease patients. *J Clin Invest* 2015; **125**: 1–8.

52 Byrne LM, Rodrigues FB, Johnson EB, *et al.* Cerebrospinal fluid neurogranin and TREM2 in Huntington's disease. *Sci Rep* 2018; **8;** 4260.

53 Thelin E, Al Nimer F, Frostell A, *et al.* A Serum Protein Biomarker Panel Improves Outcome Prediction in Human Traumatic Brain Injury. *J Neurotrauma* 2019; **36(20):** 2850-62.

54 Zeitlberger AM, Thomas-Black G, Garcia-Moreno H, *et al.* Plasma markers of neurodegeneration are raised in friedreich's ataxia. *Front Cell Neurosci* 2018; **12:** 366.

55 Byrne LM, Rodrigues FB, Johnson EB, *et al.* Evaluation of mutant huntingtin and neurofilament proteins as potential markers in Huntington's disease. *Sci Transl Med* 2018; **10(458)**.

56 van Buuren S. Flexible Imputation of Missing Data, Second Edition. 2018.

57 Lawrence AD, Sahakian BJ, Hodges JR, Rosser AE, Lange KW, Robbins TW. Executive and mnemonic functions in early Huntington's disease. *Brain* 1996; **119**: 1633–45.

58 Lawrence AD, Hodges JR, Rosser AE, *et al.* Evidence for specific cognitive deficits in preclinical Huntington's disease. *Brain* 1998; **121**: 1329–41.

59 Vaghi MM, Vértes PE, Kitzbichler MG, *et al.* Specific Frontostriatal Circuits for Impaired Cognitive Flexibility and Goal-Directed Planning in Obsessive-Compulsive Disorder: Evidence From Resting-State Functional Connectivity. *Biol Psychiatry* 2017; **81(8):** 708-717.

60 Ho AK, Sahakian BJ, Brown RG, *et al.* Profile of cognitive progression in early Huntington's disease. *Neurology* 2003; **61**: 1702–6.

61 Pavese N, Andrews TC, Brooks DJ, *et al.* Progressive striatal and cortical dopamine receptor dysfunction in Huntington's disease: A pet study. *Brain* 2003; **126**: 1127–35.

62 Swainson R, Hodges JR, Galton CJ, *et al.* Early detection and differential diagnosis of Alzheimer's disease and depression with neuropsychological tasks. *Dement Geriatr Cogn Disord* 2001; **12(4):** 265-80.

63 De Rover M, Pironti VA, McCabe JA, *et al.* Hippocampal dysfunction in patients with mild cognitive

impairment: A functional neuroimaging study of a visuospatial paired associates learning task. *Neuropsychologia* 2011; **49(7):** 2060-70.

64 Begeti F, Schwab LC, Mason SL, Barker RA. Hippocampal dysfunction defines disease onset in Huntington's disease. *J Neurol Neurosurg Psychiatry* 2016; 87**(9):** 975-81.

65 Lawrence AD, Watkins LH, Sahakian BJ, Hodges JR RT. Visual object and visuospatial cognition in Huntington's disease: implications for information processing in corticostriatal circuits. *Brain* 2000; **123:** 1349-64

66 Tabrizi SJ, Langbehn DR, Leavitt BR, *et al.* Biological and clinical manifestations of Huntington's disease in the longitudinal TRACK-HD study: cross-sectional analysis of baseline data. *Lancet Neurol* 2009; **8**: 791–801.

67 Paulsen JS, Long JD. Onset of Huntington's disease: Can it be purely cognitive? *Mov Disord* 2014; **29(11):** 1342-50.

68 Dumas EM, Versluis MJ, Van den Bogaard SJA, *et al.* Elevated brain iron is independent from atrophy in Huntington's Disease. *Neuroimage* 2012; **61(3):** 558-64.

69 Olsson B, Lautner R, Andreasson U, *et al.* CSF and blood biomarkers for the diagnosis of Alzheimer's disease: a systematic review and meta-analysis. *Lancet Neurol* 2016.**15(7):** 673-84.

70 Rodrigues FB, Byrne LM, McColgan P, *et al.* Cerebrospinal fluid inflammatory biomarkers reflect clinical severity in huntington's disease. *PLoS One* 2016; **11(9)**:e0163479.

71 von Essen MR, Hellem MNN, Vinther-Jensen T, *et al.* Early Intrathecal T Helper 17.1 Cell Activity in Huntington Disease. *Ann Neurol* 2020; **87(2)**:246-255.
